# Supplementary material for: Visualization and quantification of coral reef soundscapes using CoralSoundExplorer software
Source: PLoS Comput Biol. 2025 Apr 10;21(4):e1012050. doi: 10.1371/journal.pcbi.1012050 (PMC12017563; doi:10.1371/journal.pcbi.1012050)
Supplement: S1 Text — Complementary material to part “II. CoralSoundExplorer software: Analysis workflow, graphical output and measurable metrics”. (DOCX) [file pcbi.1012050.s001.docx]

# S1 Text: Detailed Methodology of *CoralSoundExplorer*

This supplementary material first summarizes the analysis principle of unsupervised learning which forms the core of *CoralSoundExplorer*. Then details of the *CoralSoundExplorer* workflow and of the results extraction process not provided in section II are described.

# Soundscape analysis paradigm

The analyses of sound signals proposed by *CoralSoundExplorer* are based on their projections into an initial acoustic representation space derived either from a mel-spectrum, a mel-spectrogram or a convolutional neural network (CNN) [1] trained for sound analysis. This acoustic space is assumed to be metric, i.e. where the notion of distance between data points projected into this space has a geometric meaning (for the analysis of sound recordings, the data points are points in the space where each corresponds to a sound signal of a certain duration, decided by the person conducting the analysis). Two points close in distance correspond to two sound signals sharing common acoustic characteristics, and two points further away correspond to two different sound signals. The result is that close sound signals are grouped together in the same embedding subspace and, conversely, groups of different sound signals are moved further apart. This notion of grouping by similarity in the acoustic space is that of clusters in machine learning. The relative organization of data points (sounds) in the acoustic space can be studied as regards to their labels. The aim is to find out whether the annotations previously made on each signal, based on knowledge of their recording conditions or content (such as the time and place of recording) enable signals to be grouped (and therefore classified) according to their acoustic characteristics. The relative organization of sounds in the acoustic space can be also studied using a more direct unsupervised approach, looking for the most identifiable unsupervised clusters in the embedding space. Explanations for these unsupervised clusters can be found by identifying the similarity of sounds by searching for features that allow grouping or distancing. This can then be sought directly by careful listening and viewing of the spectrogram, or by cross-referencing with labels previously assigned to the recordings. In addition to these analyses based on a categorical organization of the data set, it is also possible to follow a path in the embedding space as a function of a continuous variable value corresponding to each signal. In the context of analyzing the time characteristic of signals and observing the phenology of soundscapes, this continuous variable can be the day or hour of recording, or any other meaningful time interval.

The number of embedding dimensions directly derived from the neural network can be large, posing a problem of interpretability and calculation of inference metrics due to the curse of dimensionality [2]. Cluster extraction or analysis methods can suffer from this. Specifically, density-based cluster extraction methods, such as HDBSCAN, are known to perform poorly in high dimensions [3]. To overcome this difficulty, it is useful to use dimensionality reduction methods. The best-known is PCA, but this linear approach is often unsuitable for complex problems due to the potentially significant loss of information at low dimensions. Several nonlinear methods have been proposed to overcome this problem, such as t-SNE methods [4] or, more recently, the UMAP approach. The latter is based on a consistent mathematical approach that justifies the globally and locally conservative nature of the data structure. Thus, UMAP generally preserves the clusters present in the dataset on which it is applied and even tends to increase the clustering effect [5]. However, UMAP is often only used as an illustrative support in 2- or 3-dimensions due to the stochastic nature of its algorithmic implementation. This stochastic character, leading to different representations depending on a random seed, cannot be ignored when using UMAP to extract quantitative measurements, and its effect on projection and on the analyses that can be deduced from it needs to be addressed. One solution, which we have employed here, is to work based on several realizations of UMAP from the data and use the central tendencies to characterize and quantify the organization of sounds in the UMAP embedding.

# Signal pre-processing and the initial acoustic space

In the first step, *CoralSoundExplore*r segments each raw sound recording into one-second segments (the raw recordings in the Bora-Bora dataset were 60 seconds long). Each one-second sound segment is then projected into an acoustic space. Three different acoustic spaces can be selected: the mel-spectrum, the mel-spectrogram, and the VGGish embedding.

The mel-spectrum is derived by computing the spectrum over an entire second of sound, which is frequency-scaled using a mel scale of 64 bands. Thus each second of sound is reduced to 64 features. The final amplitudes are transformed using the natural logarithm. The mel scale ranges from a minimum to a maximum frequency, adjustable by the user. For the Bora-Bora coral reef study, these were set to 70 Hz and 2000 Hz. The mel frequency bands are calculated using Douglas’ formula [6], implemented with 64 triangular filters.

The mel-spectrogram transformation is built following the same process but it is iteratively applied over shorter time windows across the entire second to preserve temporal information. For the Bora-Bora dataset, the mel-spectrogram was computed over one hundred time steps per one second sound sample, using a 2048-sample STFT Hanning window and a hop size of 0.01s (for a sampling frequency of 44100 Hz this corresponds to a window size of 46.4 ms and a time step of 441 samples). The resulting acoustic space consists of 6400 features (i.e., the number of pixels in the mel-spectrogram).

The VGGish acoustic projection takes the first 0.96 seconds of the mel-spectrogram as input to a pre-trained convolutional neural network (CNN): the VGGish network. To ensure consistency across projections and maintain a minimal one-second analysis step, the remaining 0.04 seconds of the mel-spectrogram are excluded. The CNN maps the input to a reduced feature representation of 128 dimensions. Non-significant dimensions (those with constant values across the dataset) can be excluded because they do not carry information. For the Bora-Bora dataset, only 119 of the original 128 VGGish dimensions were retained.

For all three acoustic projections, mel-spectrum, mel-spectrogram, and VGGish embeddings, the projected datasets were robustly scaled. This involved centering on the median and scaling by the interquartile range (IQR) between the first and third quartiles to balance the contributions of each dimension before UMAP dimensionality reduction.

*CoralSoundExplorer* supports integration times longer than one second. For the coral reef recordings analyzed in this study, an integration time of 15 seconds was used. Successive sound segments were grouped by calculating the centroids of their coordinates in the embedding space. In the case of the Bora-Bora dataset, the original recordings consisted of 1305 separate 1-minute recordings, resulting in 1305 × 60 = 78300 frames per second in the acoustic space. By grouping the frames in 15-second intervals, the final dataset was reduced to 5220 data points, each representing 15 seconds of recording.

# UMAPs computation.

From the original acoustic space, *CoralSoundExplorer* calculates a given number of independent UMAP transformations (Python package UMAP-Learn version 0.5.3, https://umap-learn.readthedocs.io). Each UMAP algorithm processing differs from the initialization random seed. The metric used to compute the distance is the Manhattan distance. The number of neighbors is set to 15 and the minimum distance in the UMAP representation is set to 0.

# Dataset Analysis process

## Clusterability of predefined labels

Once the UMAPs process is completed, as previously mentioned, we propose the silhouette index to test whether predefined labels can be used to discriminate data points. The silhouette index is directly linked to the notion of topography, which lies at the very heart of clustering and UMAP transformation. The computation of the silhouette index has been shown to be compatible with UMAP transformation [7]. Conceptually, for each data point, this index is related to the ratio between the average distance separating it from other data points belonging to the same category and the average distance separating it from data points belonging to the closest category. For a category, the silhouette index is the average of all the data points in the cluster. Silhouette index values could occur, which theoretically means that sounds are assigned to the wrong category. In the case of the present use of the silhouette index, this does not really make sense. However negative values, slightly less than zero, could appear. These should be treated as if they were zero.

## Unsupervised clusters identification

To find unsupervised clusters in the dataset, we chose to use the HDBSCAN algorithm [8]. The use of UMAPs as the basis for this cluster-finding process is motivated by the fact that the UMAP process is known to increase the clusterability and therefore the HDBSCAN performance [9]. Indeed, the average Hopkins statistics [10], which assesses the clusterability of a dataset is 0.98 for the 3D UMAP used to calculate the average distance matrix, while it is 0.79 for the 119d VGGish space. The input to the HDBSCAN algorithm is the same average distance matrix used to calculate the silhouette index. The HDBSCAN implementation is that available in the HDBSCAN* Python package (version 0.8.33 [https://hdbscan.readthedocs.io](https://hdbscan.readthedocs.io/)). The minimum number of samples to consider a cluster and the minimum number of neighbors to consider a data point as a core point are by default both set to 100, which, in terms of recording duration, correspond to 25 minutes if the integration time is set to 15s. The “cluster_selection_epsilon” and “alpha” parameters, which are linked to the merging of data points into clusters, are by default set to 0 and 1 respectively. The algorithm used for final cluster selection is either the “Excess of Mass” algorithm or the ‘leaf’ algorithm. The former is expected to lead to larger and fewer clusters, while the latter is expected to give most fine grained and homogeneous clusters according to the HDBSCAN* Python package presentation (<https://hdbscan.readthedocs.io/en/latest/parameter_selection.html> accessed Nov. 13th 2023). In this way, two different clustering can be obtained at 2 different scales while the other clustering parameters remain constant.

## Temporal monitoring of the soundscape

This feature of *CoralSoundExplorer* allows to visualize temporal paths linking points representing sounds in acoustic spaces defined by 2D or 3D UMAP or PCA representations. In the example of the Bora-Bora recordings, it is for instance possible to trace these paths over the course of a day. The data points (or the sound projections) corresponding to each day recording session at each recording place are first sorted by date, from the first recording date (noon) to the last recording date (noon the following day). From the sorted data points, the coordinates of each path point were obtained using a sliding average over a one-hour frame to reduce the noise effect. The central time of the rolling mean frames is taken as the time point.

This process is applied to each of the multiple computational UMAPs to obtain a stable and reliable representation of the soundscape phenology. The distances between an average starting point and each of the path points computed on the UMAPs are then measured. For each of the three sites considered, the average starting point is computed from the coordinates of each of the start points on each of the recording days (at t = 0). This measure is related to the average distance between the starting point and its 100 first nearest neighbors. For each day and each site, *CoralSoundExplorer* retains the median of unique measurements (one per computational UMAP) of distances relative to the average starting point as a function of daytime, leading to a single stable measurement. Together with this median, the measure of the first decile and the 9th decile of the relative distance is given to have an indication on the dispersion of the 100 calculated relative distances at each point concerned.

# References

1[. Albawi S, Mohammed TA, Al-Zawi S. Understanding of a convolutional neural network. In: 2017 International Conference on Engineering and Technology (ICET). Antalya, Turkey: IEEE; 2017. p. 1‑6.](https://www.zotero.org/google-docs/?broken=k10UDb)

2[. Chen L. Curse of Dimensionality. In: Liu L, Özsu MT, editors. Encyclopedia of Database Systems. Boston, MA: Springer US; 2009. p. 545‑6.](https://www.zotero.org/google-docs/?broken=n1r07g)

3[. Musdholifah A, Hashim SZM. Cluster Analysis on High-Dimensional Data: A Comparison of Density-based Clustering Algorithms. Int J Comput Appl. 2013;66(19):22‑8.](https://www.zotero.org/google-docs/?broken=cSKgPp)

4[. van der Maaten L, Hinton G. Visualizing data using t-SNE. J Mach Learn Res. 2008;9:2579‑605.](https://www.zotero.org/google-docs/?broken=FnqmOH)

5[. Allaoui M, Kherfi ML, Cheriet A. Considerably Improving Clustering Algorithms Using UMAP Dimensionality Reduction Technique: A Comparative Study. In: Image and Signal Processing. Cham: Springer; 2020. p. 317‑25. (Lecture Notes in Computer Science; vol. 12119).](https://www.zotero.org/google-docs/?broken=gnVCwZ)

6[. O’Shaughnessy D. Speech communication: human and machine. Reading, MA: Addison-Wesley; 1987. 568 p.](https://www.zotero.org/google-docs/?broken=GiW41o)

7[. Dalmaijer ES, Nord CL, Astle DE. Statistical power for cluster analysis. BMC Bioinformatics. 2022;23(1):205.](https://www.zotero.org/google-docs/?broken=wAbMtY)

8[. Han H, Li W, Wang J, Qin G, Qin X. Enhance explainability of manifold learning. Neurocomputing. 2022;500(C):877‑95.](https://www.zotero.org/google-docs/?broken=7xGpYl)

[9. McInnes L, Healy J, Astels S. hdbscan: Hierarchical density based clustering. J Open Source Softw. 2017;2(11):205.](https://www.zotero.org/google-docs/?broken=1HxlD3)

10[. Hopkins B, Skellam JG. New Method for determining the Type of Distribution of Plant Individuals. Ann Bot. 1954;18(2):213‑27.](https://www.zotero.org/google-docs/?broken=7vsgDb)
