# Supplementary material for: Visualization and quantification of coral reef soundscapes using CoralSoundExplorer software
Source: PLoS Comput Biol. 2025 Apr 10;21(4):e1012050. doi: 10.1371/journal.pcbi.1012050 (PMC12017563; doi:10.1371/journal.pcbi.1012050)
Supplement: S2 Text — Complementary material to part “IV. CoralSoundExplorer software: A parametric study using the Bora-Bora dataset”. (DOCX) [file pcbi.1012050.s002.docx]

# S2 Text: Parametric study of CoralSoundExplorer

# Choice of the initial acoustic projection space

The different 3D UMAP visualizations for each initial acoustic space (mel-spectrogram, mel-spectrum, the VGGish embedding from 70–2000 Hz input mel–spectrograms and the VGGish embedding from 125–7500 Hz input mel–spectrograms) are shown in Fig. S2-Text-1, with points colored according to the period of the day (day or night) and the recording site. Silhouette matrix plots corresponding to these four configurations are shown in Fig. S2-Text-2, considering the predefined label combinations site/replicate/period. The VGGish 125–7500 Hz projection shows slightly more contrast between night and day for the boat site, the undisturbed site, and the first two recording days at the tourist site. The third day and night of the tourist site also appear more distinct from each other and from the other recordings. Overall, however, the choice of acoustic space has only a minor impact on the distinctions between the site/period and site/replicate/period categories.

| 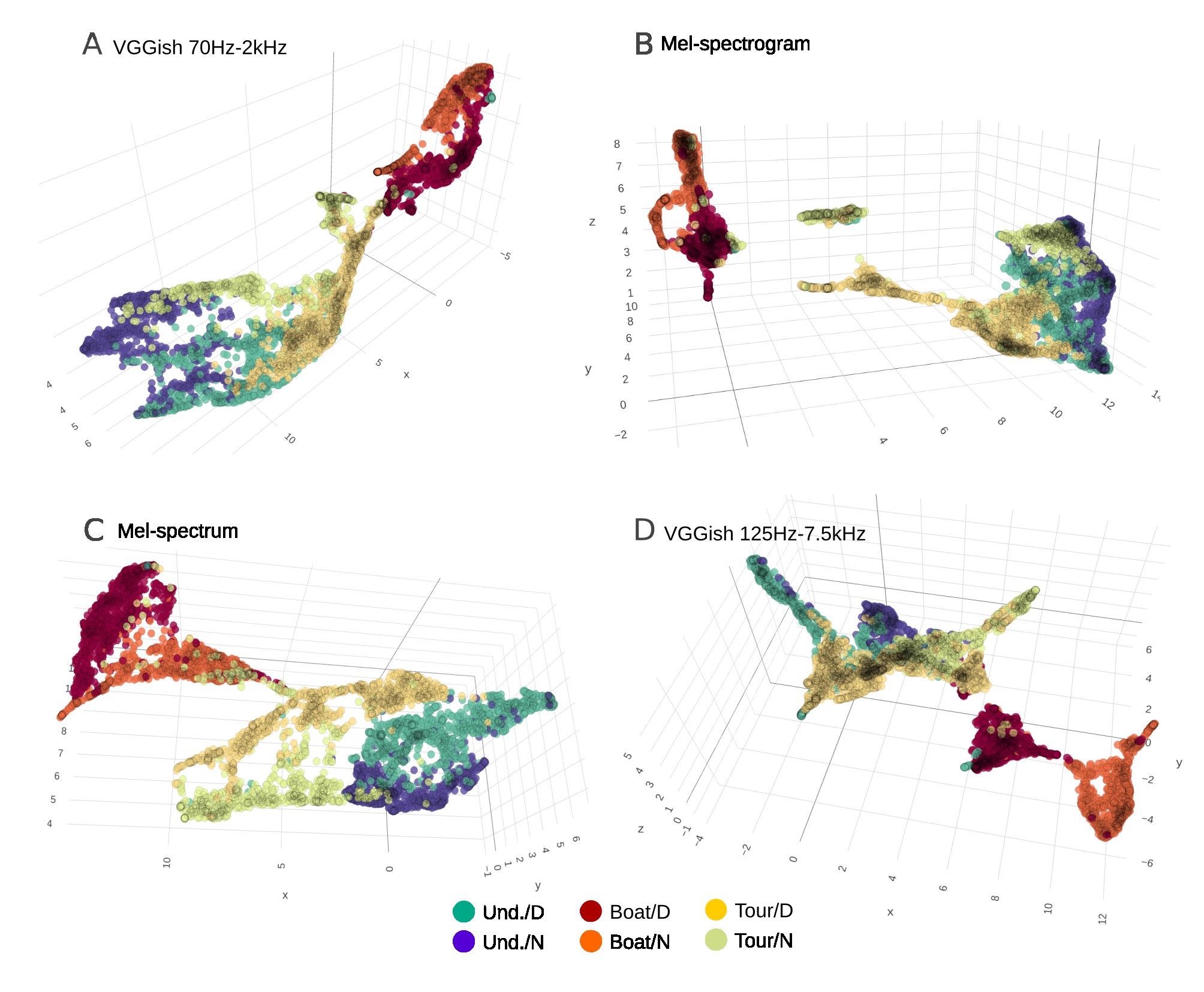 |
| --- |
| **Fig S2-Text-1:** **Three-dimensional visualization of the acoustic space of coral reef soundscapes using *CoralSoundExplorer* software (UMAP 3D view).**  Each dot corresponds to 15 seconds of sound recording. The color of each dot corresponds to a category of the composite predefined label of recording site and diurnal/nocturnal period. (A) Using VGGish 70-2kHz for the features extraction of the initial acoustic projection space. (B) Using the mel-spectrogram for the features extraction of the initial acoustic projection space. (C) Using the mel-spectrum for the features extraction of the initial acoustic projection space. (D) Using VGGish 125-7.5kHz for the features extraction of the initial acoustic projection space. |

| 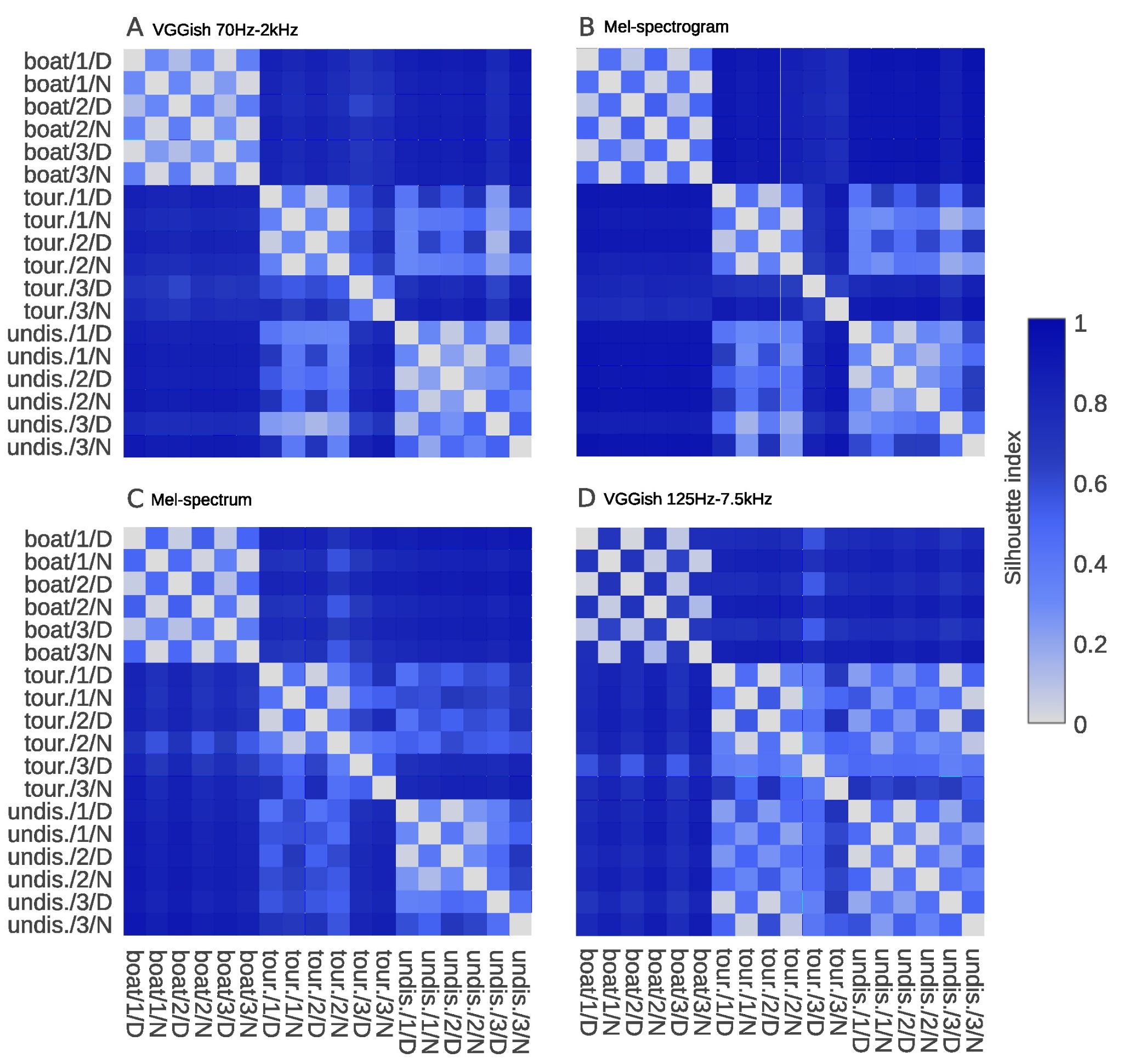 |
| --- |
| **Fig S2-Text-2: Quantification of acoustic similarity (Silhouette indices) between reef sounds recorded at Bora-Bora, considering different acoustic space projections.**  Silhouette indices are calculated from 100 UMAPs. The continuous color scale represents the index value (0: the two groups are similar, signifying homogeneous soundscapes; 1: the two groups are completely dissimilar). Recordings are labeled by site, day/night period (D: day, N: night) and replicate number (3 replicates, corresponding to 3 non-consecutive 24-hour recording periods). (A) Using VGGish 70-2kHz for the features extraction of the initial acoustic projection space. (B) Using the mel-spectrogram for the features extraction of the initial acoustic projection space. (C) Using the mel-spectrum for the features extraction of the initial acoustic projection space. (D) Using VGGish 125-7.5kHz for the features extraction of the initial acoustic projection space. |

For each initial acoustic projection space, the results of unsupervised clustering using the leaf clustering method are presented in Fig. S2-Text-3. Fig. S2-Text-4 exposes the corresponding contingency matrices comparing the unsupervised clusters with the site/replicate/period labels. All four acoustic spaces produce consistent clustering results. For example, the three daytime periods at the boat site are clustered together, as are the three nighttime periods. However, subtle differences emerge. For the VGGish 70–2000 Hz projection, daytime periods at the boat site are split into two clusters, reflecting different levels of boat noise. This split does not occur with the other projections. At the tourist site, diurnal and nocturnal periods from the first two recording days are clustered together with corresponding periods from the undisturbed site when using the VGGish 70–2000 Hz projection. In contrast, the mel-spectrum and mel-spectrogram projections cluster the two sites separately. For the VGGish 125–7500 Hz projection, some recordings from the first two days at the undisturbed site are placed into unique clusters, distinguishing these days and nights from others. This differentiation is not observed with the VGGish 70–2000 Hz projection.

| 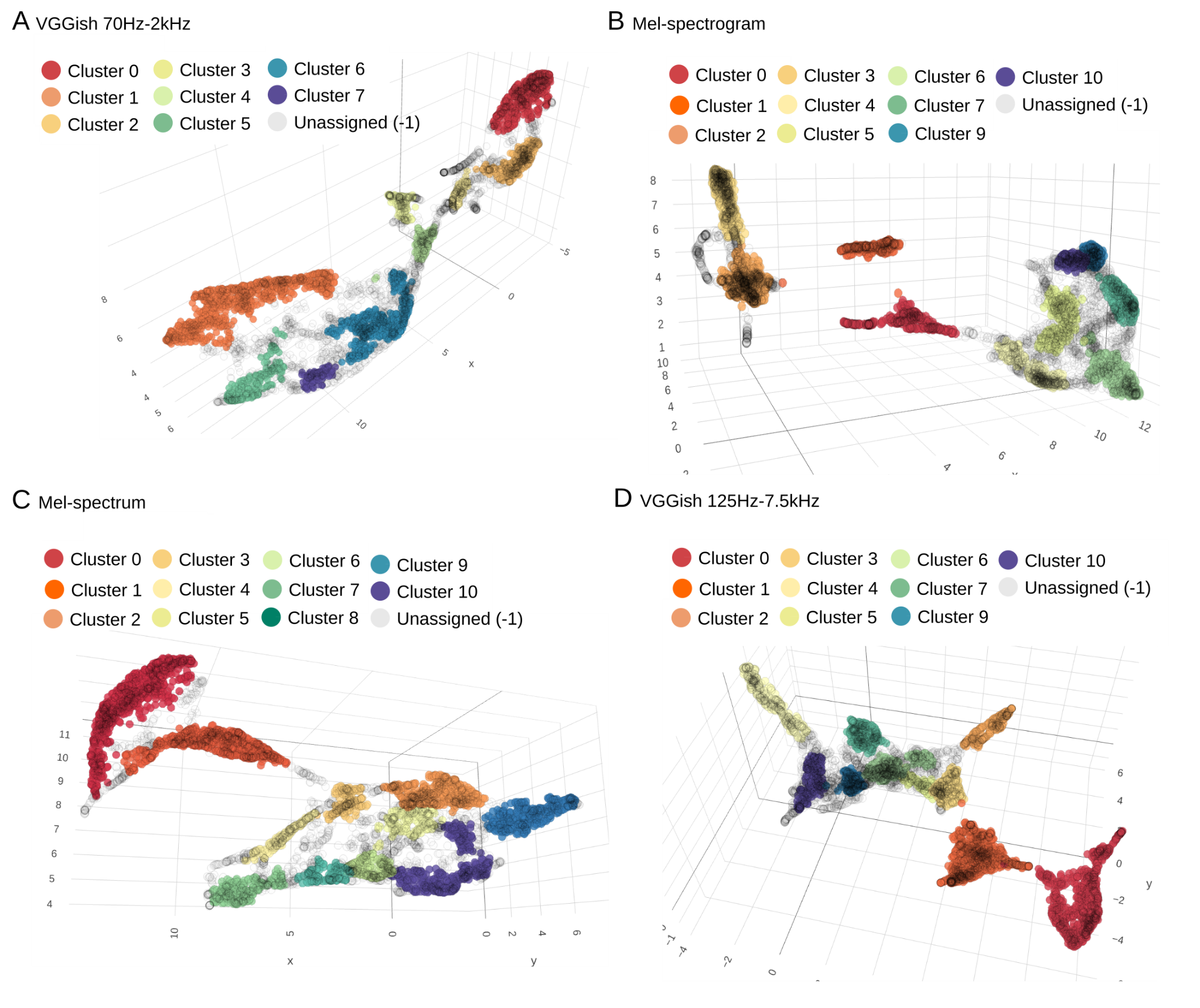 |
| --- |
| **Fig S2-Text-3:** **Unsupervised clustering of soundscapes recorded on the coral reefs of Bora-Bora, considering different acoustic space projections (UMAP 3D view).**  3D UMAP visualization using HDBSCAN with the Leaf clustering method and a minimum cluster size of 100 samples. (A) Using VGGish 70-2kHz for the features extraction of the initial acoustic projection space. (B) Using the mel-spectrogram for the features extraction of the initial acoustic projection space. (C) Using the mel-spectrum for the features extraction of the initial acoustic projection space. (D) Using VGGish 125-7.5kHz for the features extraction of the initial acoustic projection space. |

| 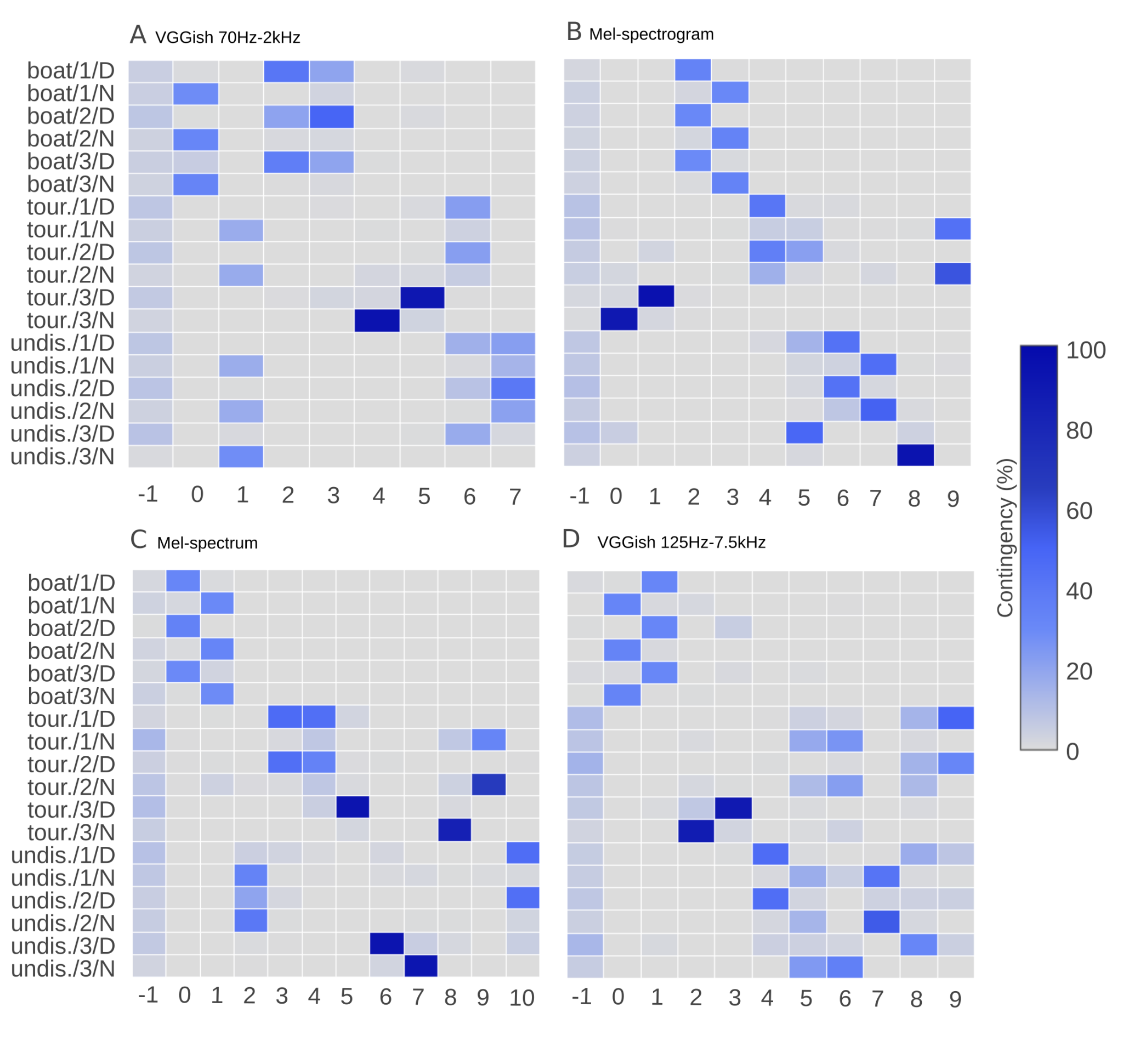 |
| --- |
| **Fig S2-Text-4: Unsupervised clustering of soundscapes recorded on the coral reefs of Bora-Bora, considering different acoustic space projections: Contingency matrix of the different categories of the composite label site/replica/period within each unsupervised cluster.**  Using HDBSCAN with the Leaf clustering method and a minimum cluster size of 100 samples. (A) Using VGGish 70-2kHz for the features extraction of the initial acoustic projection space. (B) Using the mel-spectrogram for the features extraction of the initial acoustic projection space. (C) Using the mel-spectrum for the features extraction of the initial acoustic projection space. (D) Using VGGish 125-7.5kHz for the features extraction of the initial acoustic projection space. |

Temporal monitoring results for the undisturbed site using the four acoustic projections are shown in Fig. S2-Text-5. These results indicate that the choice of acoustic space does not strongly influence conclusions. Across all projections, the first two days follow similar patterns, while the third day diverges, especially during a rain event. The relative amplitude of this deviation due to the rain event can be estimated by the ratio of the relative distances value at the peak of this deviation by its starting time value. For the mel-spectrogram and the VGGish 70–2000 Hz projections this relative amplitude is around 5 while for the mel-spectrum it is around 2 and around 3 for the VGGish 125-7500 Hz.

| 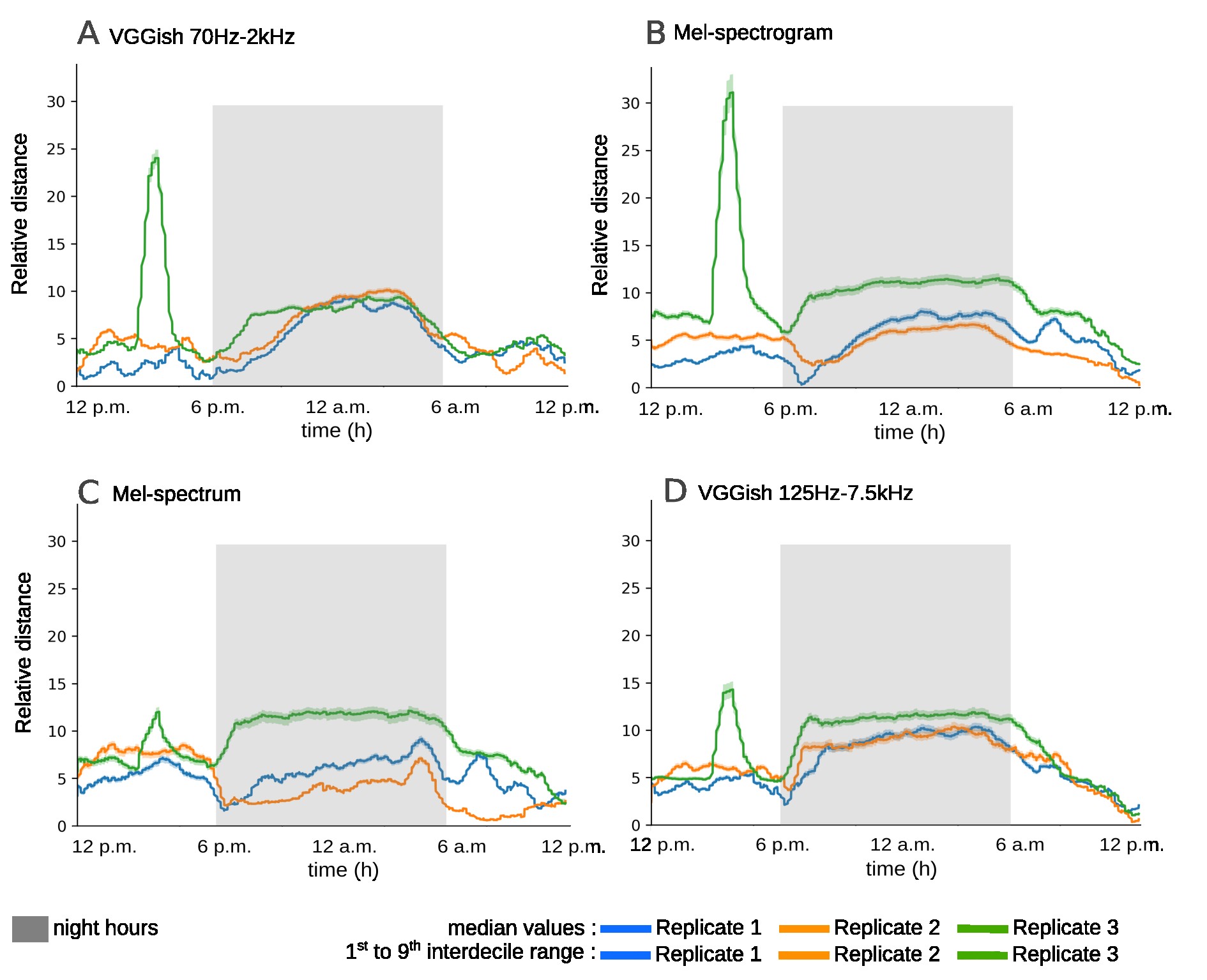 |
| --- |
| **Fig S2-Text-5: Time trajectories of the three replicates for the undisturbed site, considering different acoustic space projections.**  (A) Using VGGish 70-2kHz for the features extraction of the initial acoustic projection space. (B) Using the mel-spectrogram for the features extraction of the initial acoustic projection space. (C) Using the mel-spectrum for the features extraction of the initial acoustic projection space. (D) Using VGGish 125-7.5kHz for the features extraction of the initial acoustic projection space. |

# Choice of the dimension reduction process

In this parametric study, we compare two dimension reduction methods, UMAP and PCA, and the use of direct VGGish 70–2000 Hz features as inputs for distance matrix computation. These PCA and direct embedding (i.e. no dimension reduction) options are hidden in *CoralSoundExplorer.* The direct VGGish embedding is obtained by setting the UMAPs dimensionality to 0. The PCA is obtained by setting a negative number of UMAPs dimensions; the absolute value of this number corresponds to the PCA dimensionality. Here, both UMAP and PCA were applied to reduce the dimensionality to three dimensions, with UMAP repeated 100 times for averaging. The resulting 3D plots from UMAP and PCA are displayed in Fig. S2-Text-6. Only from this figure, it seems that the 3D UMAP embedding leads to a better separability than the 3D PCA of points according to the composite predefined label site/period.

| 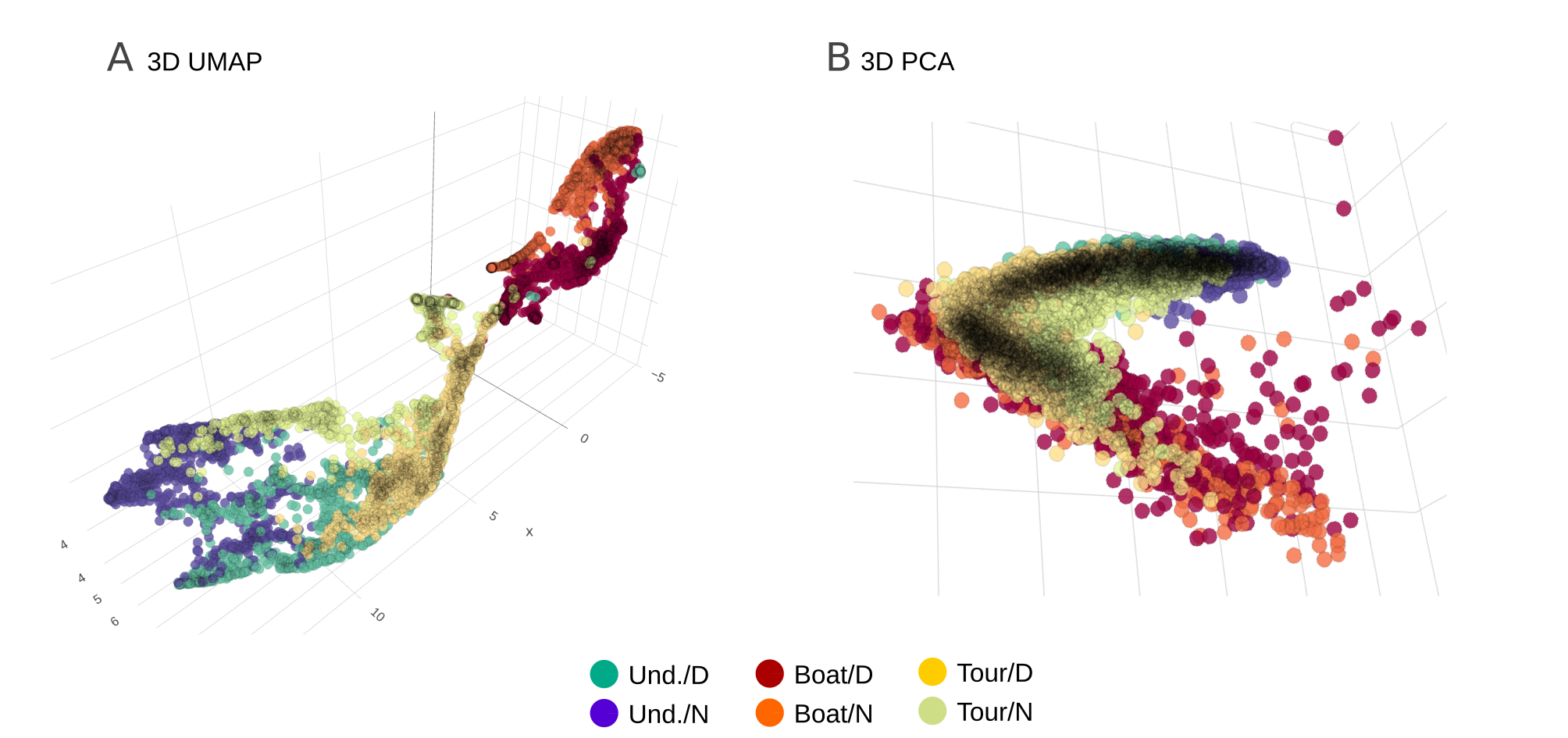 |
| --- |
| **Fig S2-Text-6:** **Three-dimensional visualization of the acoustic space of coral reef soundscapes using *CoralSoundExplorer* software.**  Each dot corresponds to 15 seconds of sound recording. The color of each dot corresponds to a category of the composite predefined label of recording site and diurnal/nocturnal period. (A) Using 3D UMAPs. (B) Using 3D PCA. |

The silhouette matrices derived from the predefined cluster analysis are shown in Fig. S2-Text-7. Comparing the 3D UMAP, 3D PCA, and direct VGGish 70–2000 Hz approaches, the silhouette values show greater differentiation between the boat site and the other two sites when using UMAP. Differences between diurnal and nocturnal periods at the boat site are also more pronounced with UMAP. For the undisturbed site, the distinctions between days and nights, as well as comparisons between the undisturbed site and the tourist site, are similar across the three approaches. However, the third recording day and night at the tourist site appear more distinct from the other periods at this site when using 3D UMAP.

| **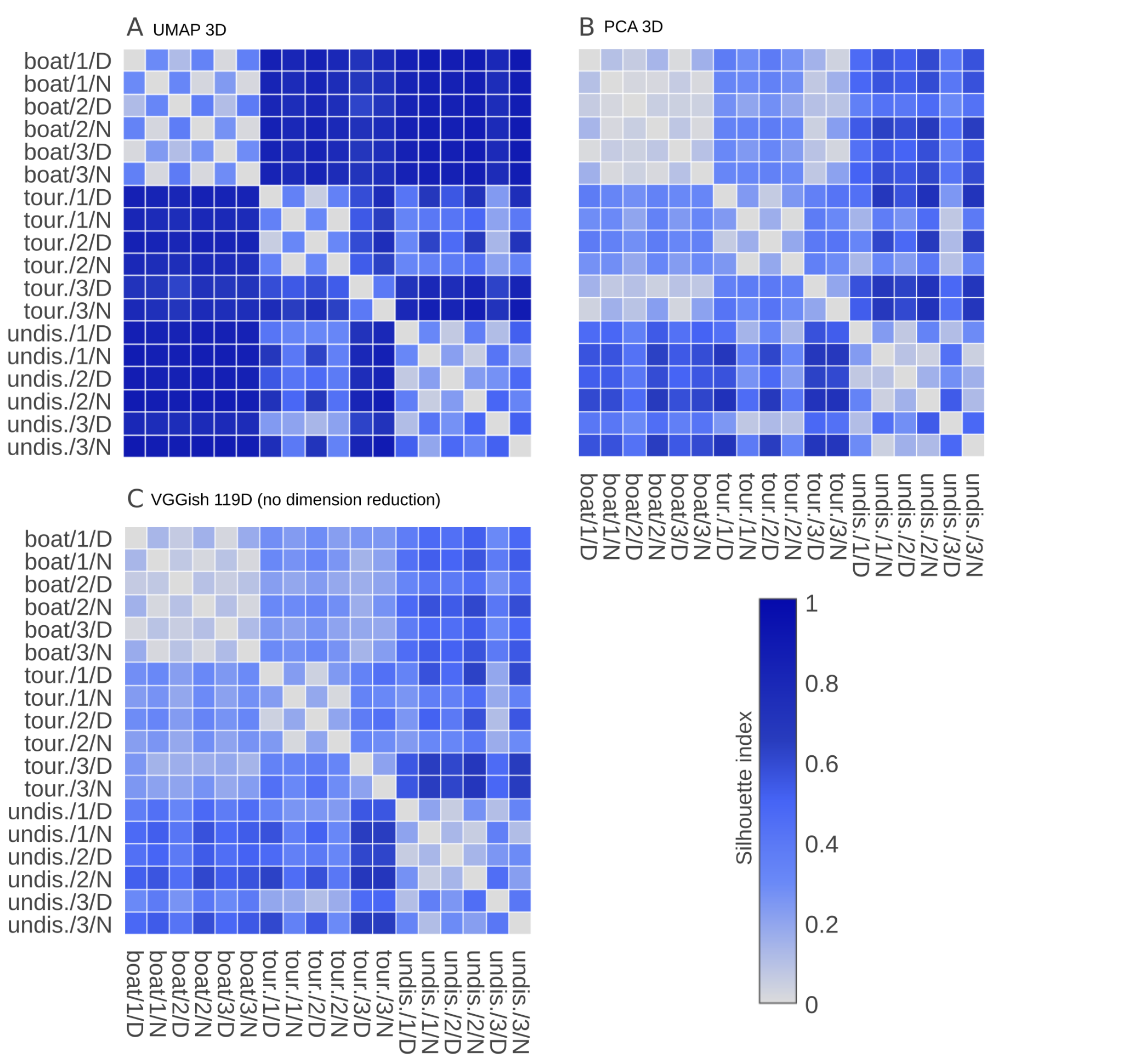** |
| --- |
| **Fig S2-Text-7: Quantification of acoustic similarity (Silhouette indices) between reef sounds recorded at Bora-Bora, considering different dimension reduction methods.**  Silhouette indices are calculated from 100 UMAPs. The continuous color scale represents the index value (0: the two groups are similar, signifying homogeneous soundscapes; 1: the two groups are completely dissimilar). Recordings are labeled by site, day/night period (D: day, N: night) and replicate number (3 replicates, corresponding to 3 non-consecutive 24-hour recording periods). (A) Using 3D UMAPs. (B) Using 3D PCA. (C) Using directly the VVGish 70-2kHz i.e. without dimension reduction process. |

The results of unsupervised cluster analysis for each dimension reduction method are presented in Fig. S2-Text-8 and Fig. S2-Text-9. The number of clusters identified with PCA and without dimension reduction is significantly reduced compared to UMAP, yielding only 3 and 2 clusters, respectively. For 3D PCA, the boat site and the third replicate of the tourist site are grouped into one cluster. The diurnal periods of the first two replicates at the tourist site and the diurnal periods of the undisturbed site form another cluster, while the nocturnal periods of the undisturbed site comprise the remaining cluster. When directly using VGGish embedding, one cluster includes only the nocturnal periods of the boat site, while the other groups samples from the first two replicates of the tourist site and the undisturbed site, without distinguishing between days and nights.

| **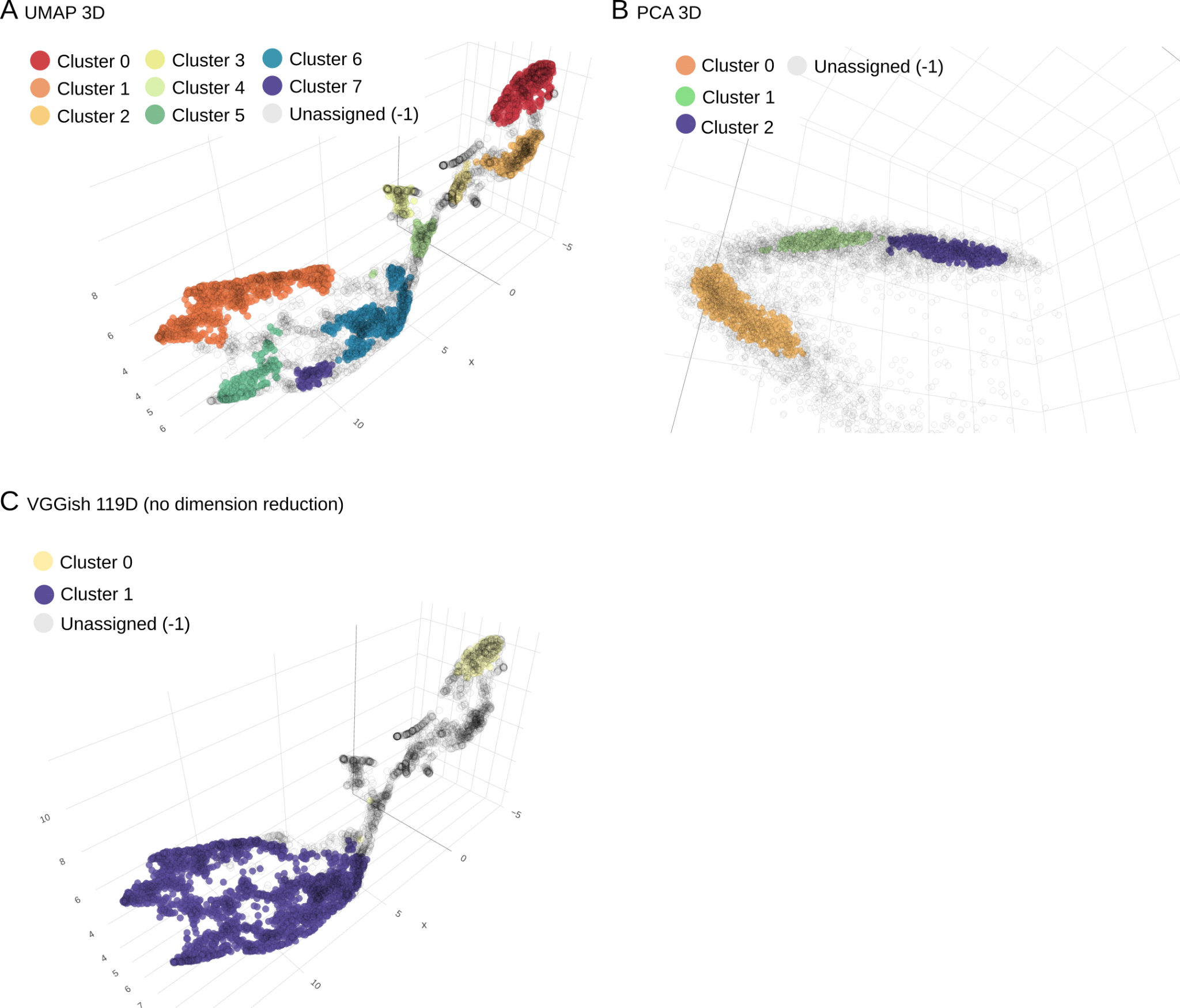** |
| --- |
| **Fig S2-Text-8:** **Unsupervised clustering of soundscapes recorded on the coral reefs of Bora-Bora, considering different dimension reduction methods.**  3D UMAP or 3D PCA visualization using HDBSCAN with the Leaf clustering method and a minimum cluster size of 100 samples. (A) Using 3D UMAPs. (B) Using 3D PCA. (C) Using directly the VVGish 70-2kHz i.e. without dimension reduction process. |

| **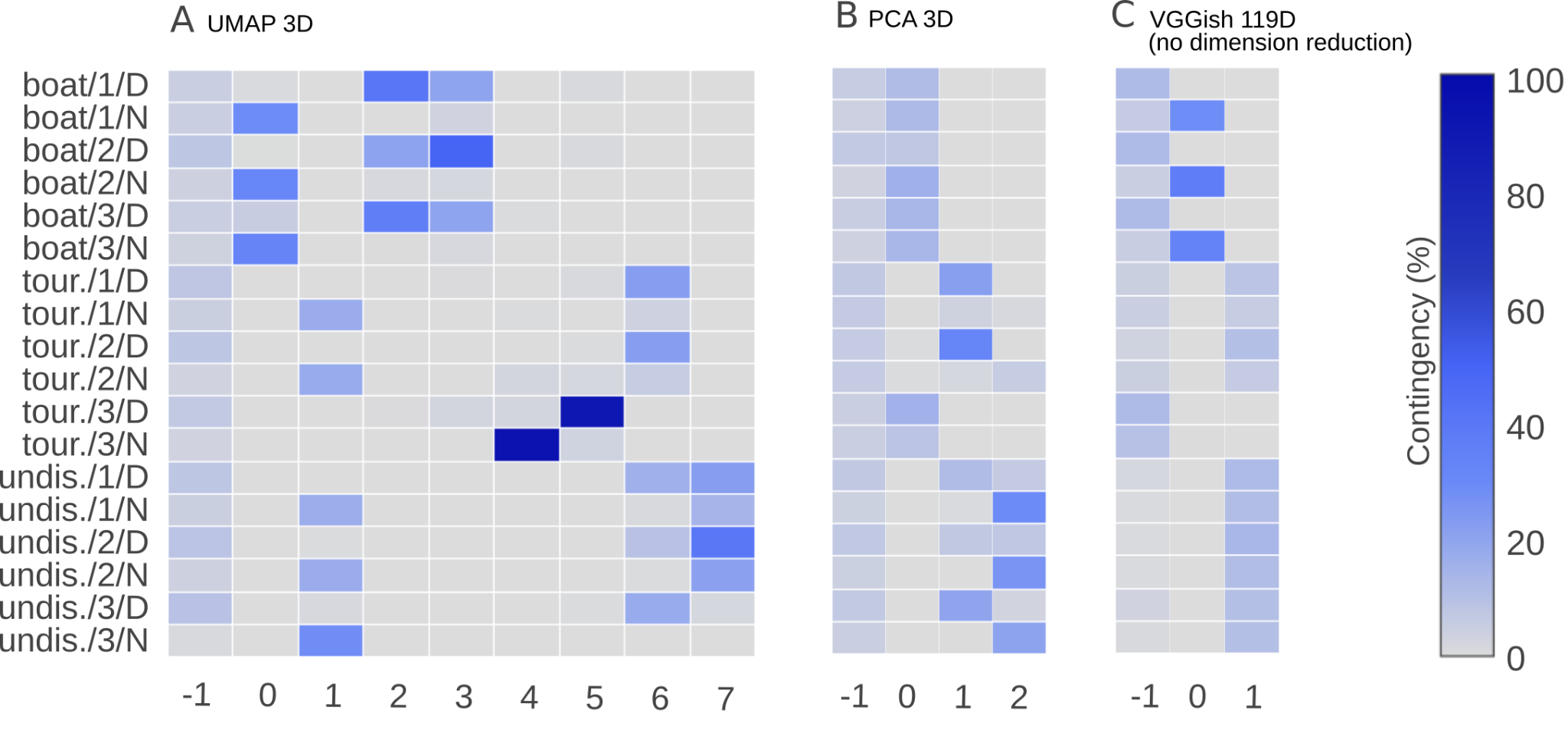** |
| --- |
| **Fig S2-Text-9: Unsupervised clustering of soundscapes recorded on the coral reefs of Bora-Bora, considering different dimension reduction methods. : Contingency matrix of the different categories of the composite label SITE/REPLICA/PERIOD within each unsupervised cluster.**  Using HDBSCAN with the Leaf clustering method and a minimum cluster size of 100 samples. (A) Using 3D UMAPs. (B) Using 3D PCA. (C) Using directly the VVGish 70-2kHz i.e. without dimension reduction process. |

The temporal dynamics of soundscapes at the undisturbed site over the three recorded days are shown in Fig. S2-Text-10. Qualitatively, these results are consistent across the three approaches. The first two days exhibit similar dynamics, while the third day diverges during the rain event. For the UMAP 3D the relative amplitude of this divergence is around 5 while for the 3D PCA or the VGGish embedding they are around 3.

| **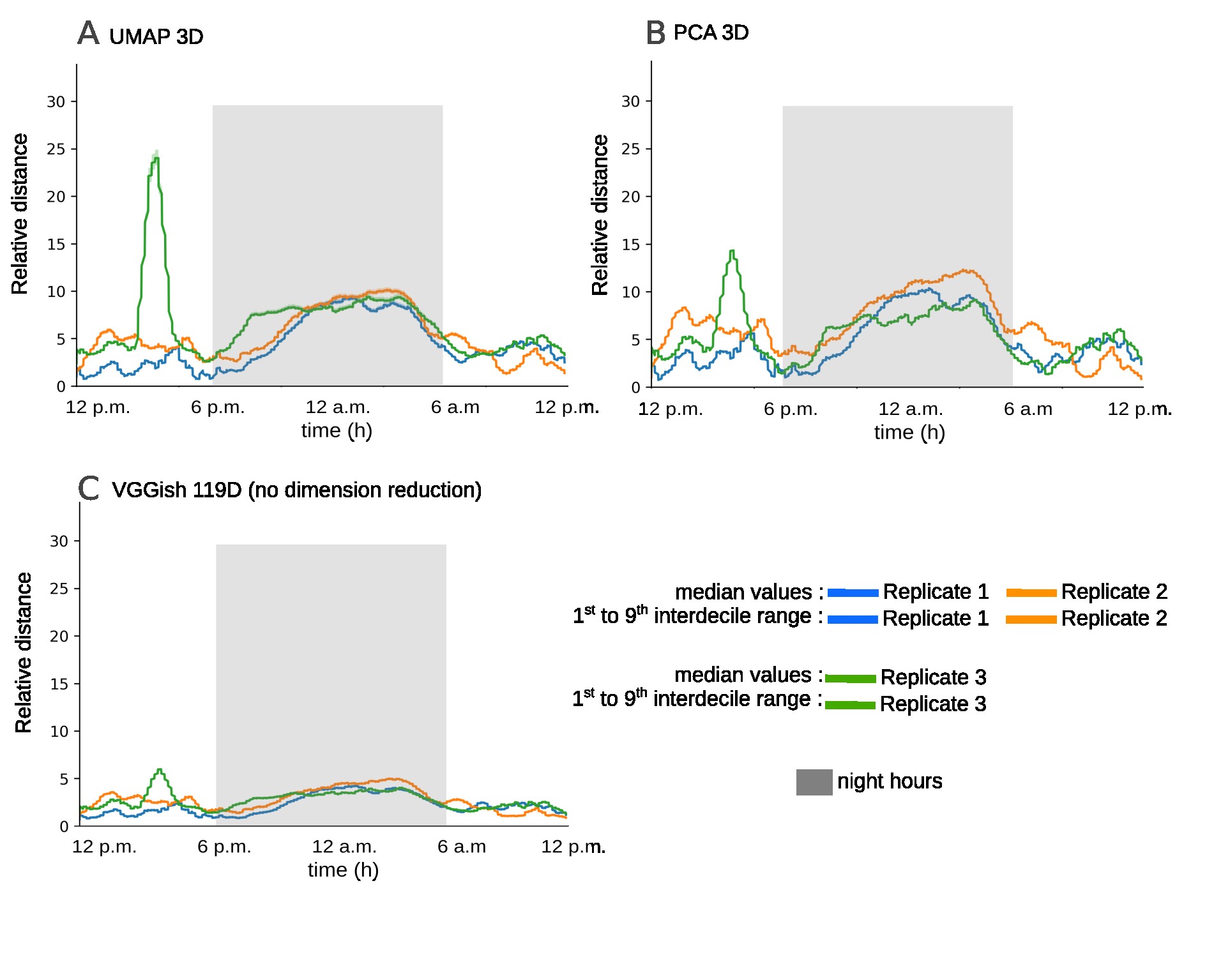** |
| --- |
| **Fig S2-Text-10: Time trajectories of the three replicates for the undisturbed site, considering different dimension reduction methods.**  (A) Using 3D UMAPs. (B) Using 3D PCA. (C) Using directly the VVGish 70-2kHz i.e. without dimension reduction process. |

# Choice of UMAPs parameters

## Number of independent UMAP computations

### Convergence of the average distance matrix

The convergence of the average distance matrix according to the number of successive unique distance matrices was evaluated considering the mean and the maximum absolute pairwise differences obtained from the average distance matrices made from *n* and *n+1* UMAPs realisations. Due to the symmetric property of the distance matrices and the zero values in their diagonals, only the lower (or upper) triangle of these matrices without their diagonals was used. The formula for these metrics is as follows:

$RelativeMeanAbsDiff\left( n \right)=\frac{\frac{2}{N\left( N-1 \right)}\sum_{i=1}^{N} \sum_{j<i} \left| D\left( n \right)_{i,j}-D\left( n-1 \right)_{i,j} \right|}{\frac{2}{N\left( N-1 \right)}\sum_{i=1}^{N} \sum_{j<i} D\left( n \right)_{i,j}}$ (1)

$RelativeMaxAbsDiff\left( n \right)=\frac{max\left\{ \left| D\left( n \right)_{i,j}-D\left( n-1 \right)_{i,j} \right| \right\}}{\frac{2}{N\left( N-1 \right)}\sum_{i=1}^{N} \sum_{j<i} D\left( n \right)_{i,j}}$ (2)

where D(n)_i,j_ are the values at row *i* and column *j* of the average distance matrix made from *n* unique matrix distances, and *N* is the total number of rows or columns of the distance matrix. The number of realizations (equal to the number of distance matrices used for averaging) varied from 1 to 100, and the final number of dimensions for the UMAP computation was either 119, 5, 3 or 2.

The results are shown in Fig. S2-Text-11. For each UMAP dimensionality, the mean relative absolute difference as a function of *n* decreases. The mean absolute relative difference between the average distance matrices obtained from 100 and 99 distance matrices is close to 0.05%, whereas the same metric obtained from the first and second distance matrices was greater than 1%, about 2% for the 119D UMAPs and 5D UMAPs and around 5% and 10% for the 3D UMAPs and the 2D UMAPs. If we consider the maximum relative error curves, the decay rates also appear to be the same whatever the UMAPs dimensionality, with a factor of about 50. However the starting value and the end values of these curves are not the same. The 119D UMAPs configuration has a maximum absolute deviation of more than 1000 for n=1 and of around 10 for n=100. The other UMAPs dimensionalities give maximum absolute deviation values around 100 or 50 for n=1 and of around 1 for n=100.

| 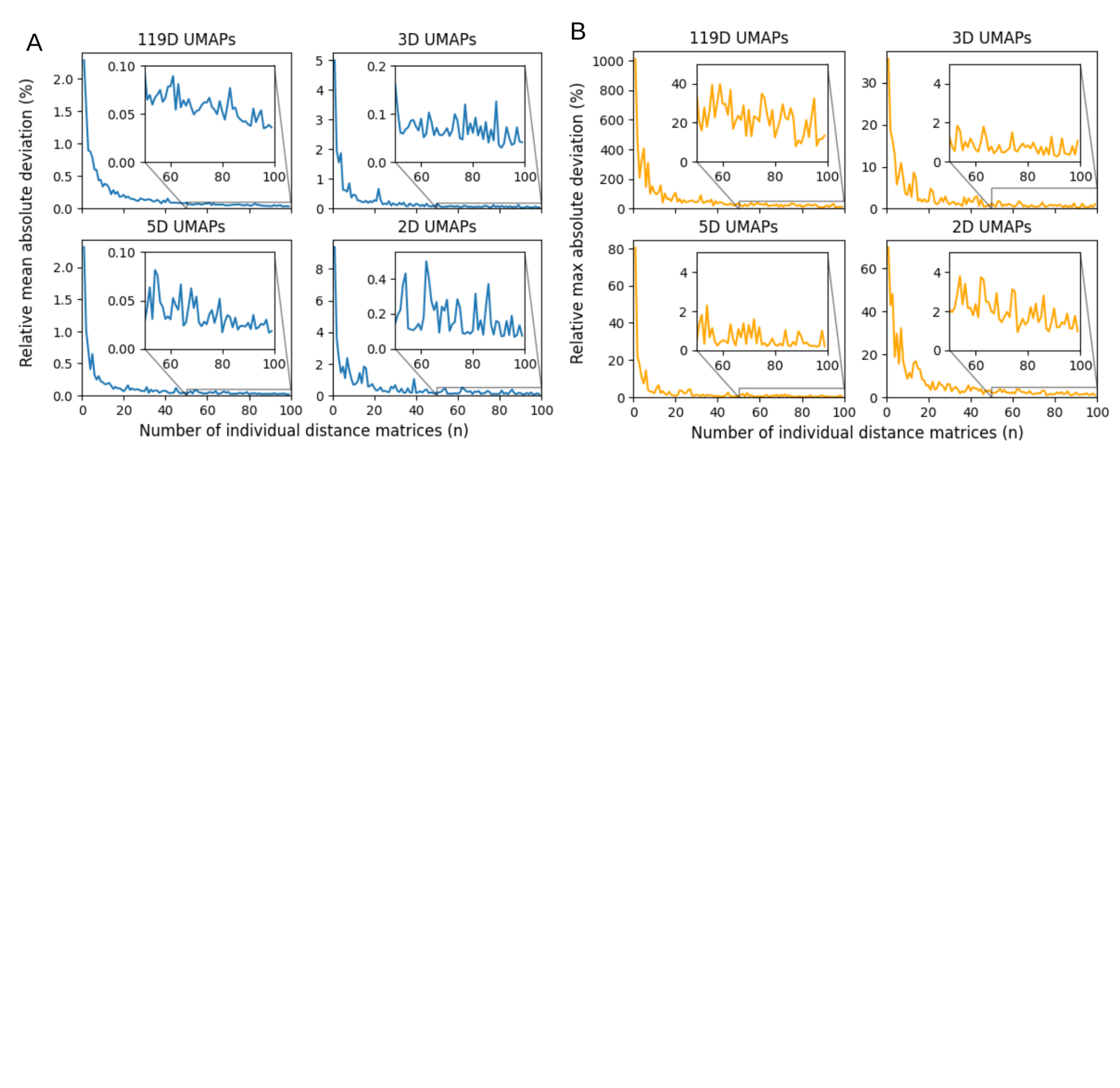 |
| --- |
| **Fig S2-Text-11:** **Absolute differences between distance matrices computed from n-1 UMAPs and n UMAPs.**  Absolute differences between distance matrices computed from n-1 UMAPs and n UMAPs relative to the mean distance observed in the distance matrix computed from n UMAPs. (A) Mean absolute differences. (B) Maximum absolute differences. |

###

### IQM metric

The results of the interquartile range of the pairwise distances divided by their median (IQM) are shown in Fig. S2-Text-12. It appears that the pairwise distances in the original VGGish embedding are more concentrated than in any mean distance matrix given by one of the three UMAP transforms, the IQM from the original VGGish is about 0.6 while the IQM of the distance matrices obtained after the UMAPs process are between 1.2 and 1.4. Considering the UMAPs process and the averaging over *n* distance matrices, it can be seen that the IQM is rapidly stable when the number of unique distance matrices increases. The low dimensional UMAPs (2D and 3D) give a higher IQM value of about 0.1 than the two higher dimensional UMAPs.

| **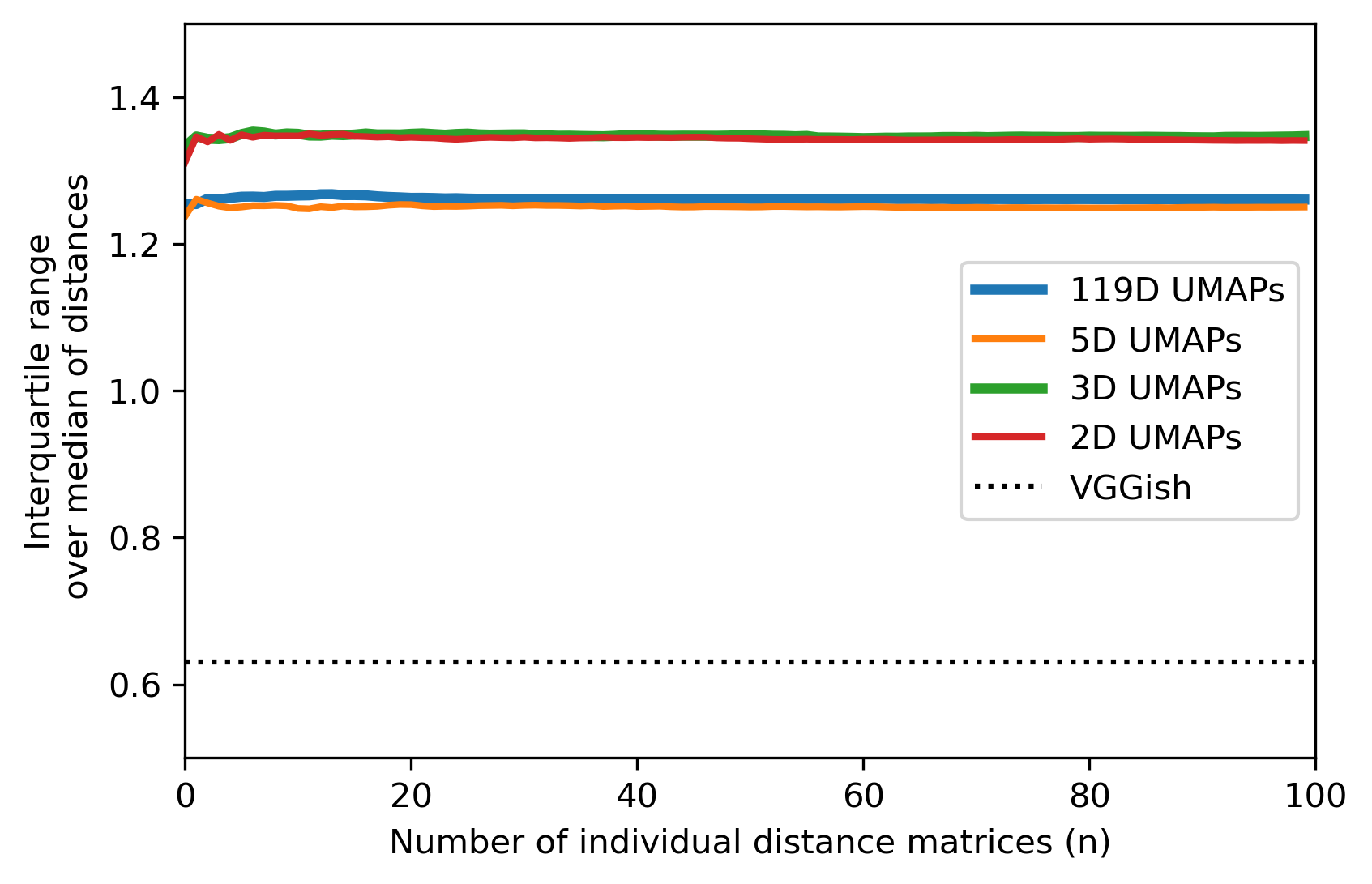** |
| --- |
| **Fig S2-Text-12:** **Interquartile range over median of all the pairwise distances between points from the original VGGish embedding or the mean distances matrix obtained from the n UMAPs.** |

## Number of UMAPs dimensions

### DLP metric

The DLP metric measures the average rate of nearest neighbors retained after dimension reduction on a number of *n* points [1]. This metric depends on the number of neighbors considered *k* and is obtained from equation (3). Where *knn_UMAP i_* are the nearest neighbors of point *i* in the UMAP embedding and *knn_VGGish i_* are the nearest neighbors of the same point in VGGish space.

$DLP\left( k \right)=\frac{\frac{1}{n}\sum_{i=1}^{n} \left| {knn}_{{UMAP}_{i}}\cap{knn}_{{VGGish}_{i}} \right|}{k}$ (3)

Given the small size of the coral reef dataset used here, this metric could be calculated using the total number of data points *N* as *n*, which would avoid the stochastic aspect due to the choice of data points *i*. This metric must be evaluated in relation to its chance level value, which depends on the number *k* relating to the number of data points in the entire data set. This chance level value is given by equation (4). We can also use a Monte-Carlo simulation, especially when *N* is large, as binomial coefficients are difficult to compute in such cases. This Monte-Carlo simulation consists of random permutations of data point positions in the original or final projection space before the distance matrix computation, and then calculating the DLP(k).

${DLP}_{0}\left( k,N \right)=\sum_{l=0}^{k} \frac{\left( k l \right)\left( \left( N-1 \right)-k k-l \right)}{\left( k \left( N-1 \right) \right)}\frac{l}{k}$ (4)

Fig. S2-Text-13 shows the DLP and the DLP rectified from its ‘by chance’ value as a function of the number of nearest neighbors considered for the 4 UMAPs dimensionality values (119,5,3,2). DLP is strongly influenced by the number of nearest neighbors considered, but the effect of the dimensionality is marginal. The maximum values of the rectified DLP curves and their approximate averages are 0.66 and 0.39 for 119d, 5d and 3d UMAPs and 0.65 and 0.39 for 2D UMAPs.

| **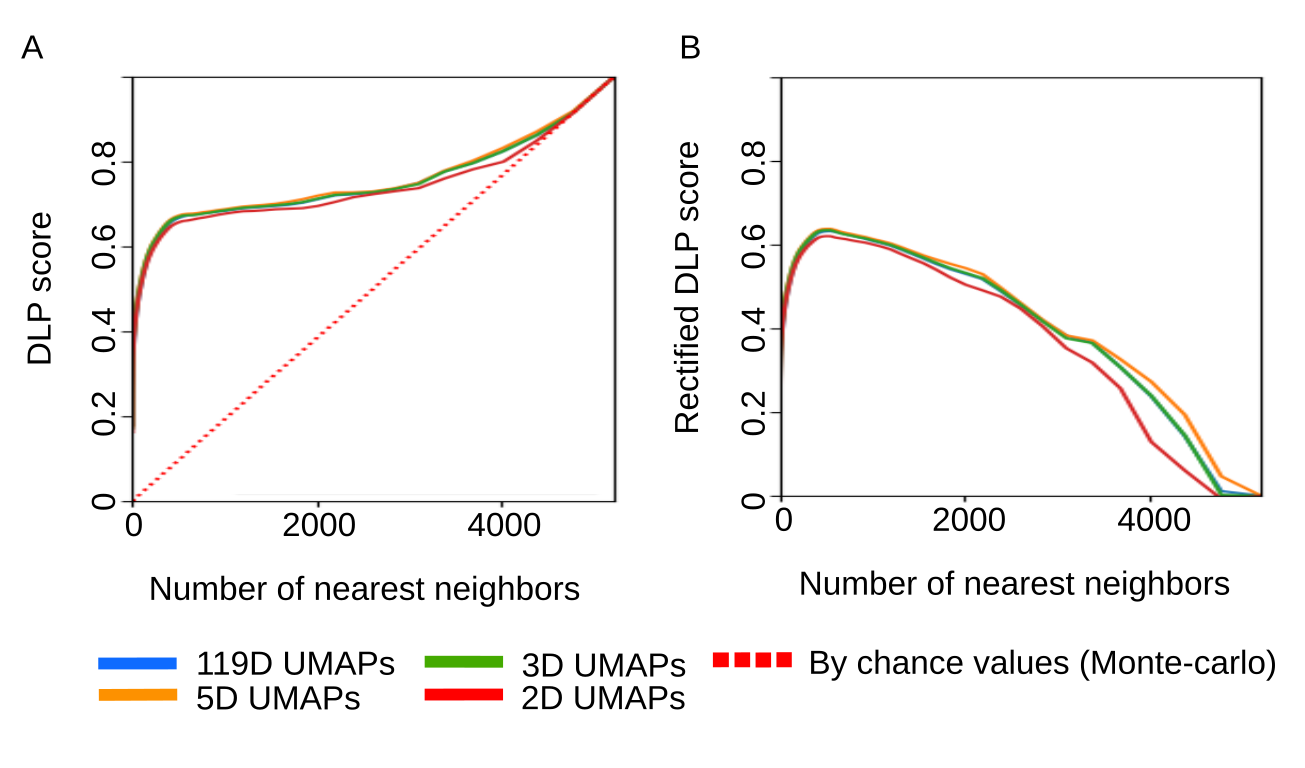** |
| --- |
| **Fig S2-Text-13:** **Local topography preservation metric of the mean average distance matrices computed from UMAPs as a function of the number of nearest neighbors for all projected data points.**  (A) Degree of Local Preservation (DLP) and (B) its rectified value by its 'by chance' value. |

###

### RTA metric

The RTA metric considers random triplets of data points and measures the ratio of these triplets whose inter data point distances ranking are the same in the original space and in the projection space [2]. This metric is stochastic, and a large number of triplets must be used to make it converge. The chance level of the RTA is equal to the number of permutations achieving the correct ranking of the 3 distances (only 1 permutation of the 3 distances is correct) out of the total number of possible permutations of the 3 distances, i.e. 1/*P*(3,3) = *(*3*!)^-1^* = 1/6.

Fig. S2-Text-14 shows the RTA scores as a function of UMAP dimensionality and its ‘by chance’ value. The number of triplets used for each average distance matrix was set at 1000, and 1000 RTA processes were performed to compute the boxplots in this figure. A one-way ANOVA performed on this dataset indicated a significant difference related to UMAP dimensionality: *F*(3.996) = 15.8; *p*<0.001. Tukey's HSD pairwise group comparisons tests yielded p-values greater than 0.01 only between 119D UMAPs and 5D UMAPs and between 3D UMAPs and 2D UMAPs. These tests also indicated that the two UMAPs with the highest dimension performed better in the sense of RTA. However, this effect is small even if statistically significant, the difference between the RTA values for two UMAP configurations of different dimensionality being around 0.01%.

| **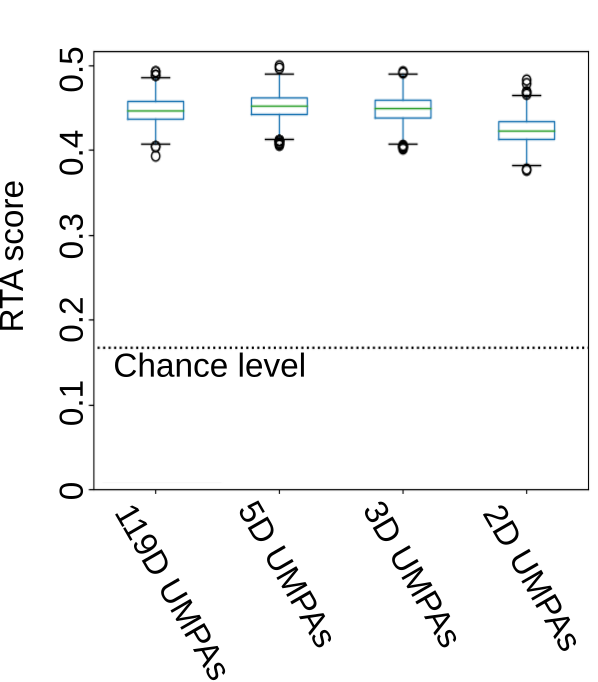** |
| --- |
| **Fig S2-Text-14:** **Global topography preservation metric of the mean average distance matrices.**  Global topography preservation metric of the mean average distance matrices computed from UMAPs (Random Triplets Accuracy (RTA) and its 'by chance' value). The boxplots were drawn from 1000 independent RTA computations for each UMAPs dimensionality. Each unique RTA value was computed from 1000 triplets. |

###

### Results given by CoralSoundExplorer

The silhouette index matrices derived from the averaged distance matrix, used for analyzing the predefined labels in *CoralSoundExplorer* across different dimensions of the 100 UMAP computations, are shown in Fig. S2-Text-15. The sample colorings, reflecting their assignment in the unsupervised cluster analysis, are presented in Fig. S2-Text-16. Additionally, the relative time trajectory plots for the undisturbed site, separated by recording day, are displayed in Fig. S2-Text-17.

The figures comparing the UMAPs dimensionality look almost identical. This emphasizes that the dimensionality of the UMAPs has no noticeable impact on the final results produced by *CoralSoundExplorer*.

| 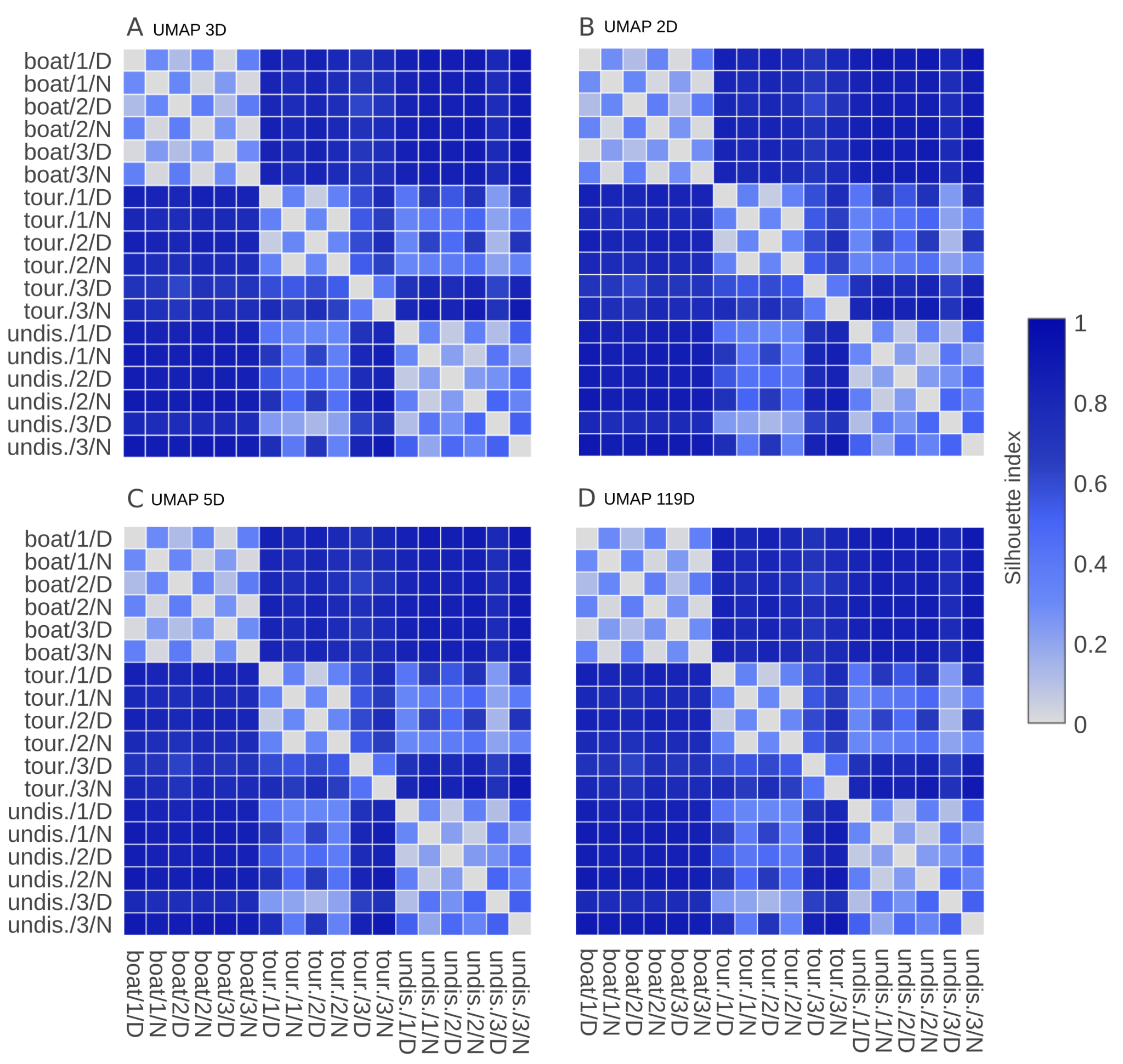 |
| --- |
| **Fig S2-Text-15: Quantification of acoustic similarity (Silhouette indices) between reef sounds recorded at Bora-Bora, considering different UMAPs dimensionalities.**  Silhouette indices are calculated from 100 UMAPs. The continuous color scale represents the index value (0: the two groups are similar, signifying homogeneous soundscapes; 1: the two groups are completely dissimilar). Recordings are labeled by site, day/night period (D: day, N: night) and replicate number (3 replicates, corresponding to 3 non-consecutive 24-hour recording periods). (A) Using 3D UMAPs. (B) Using 2D UMAPs. (C) Using 5D UMAPs. (D) Using 119D UMAPs. |

| 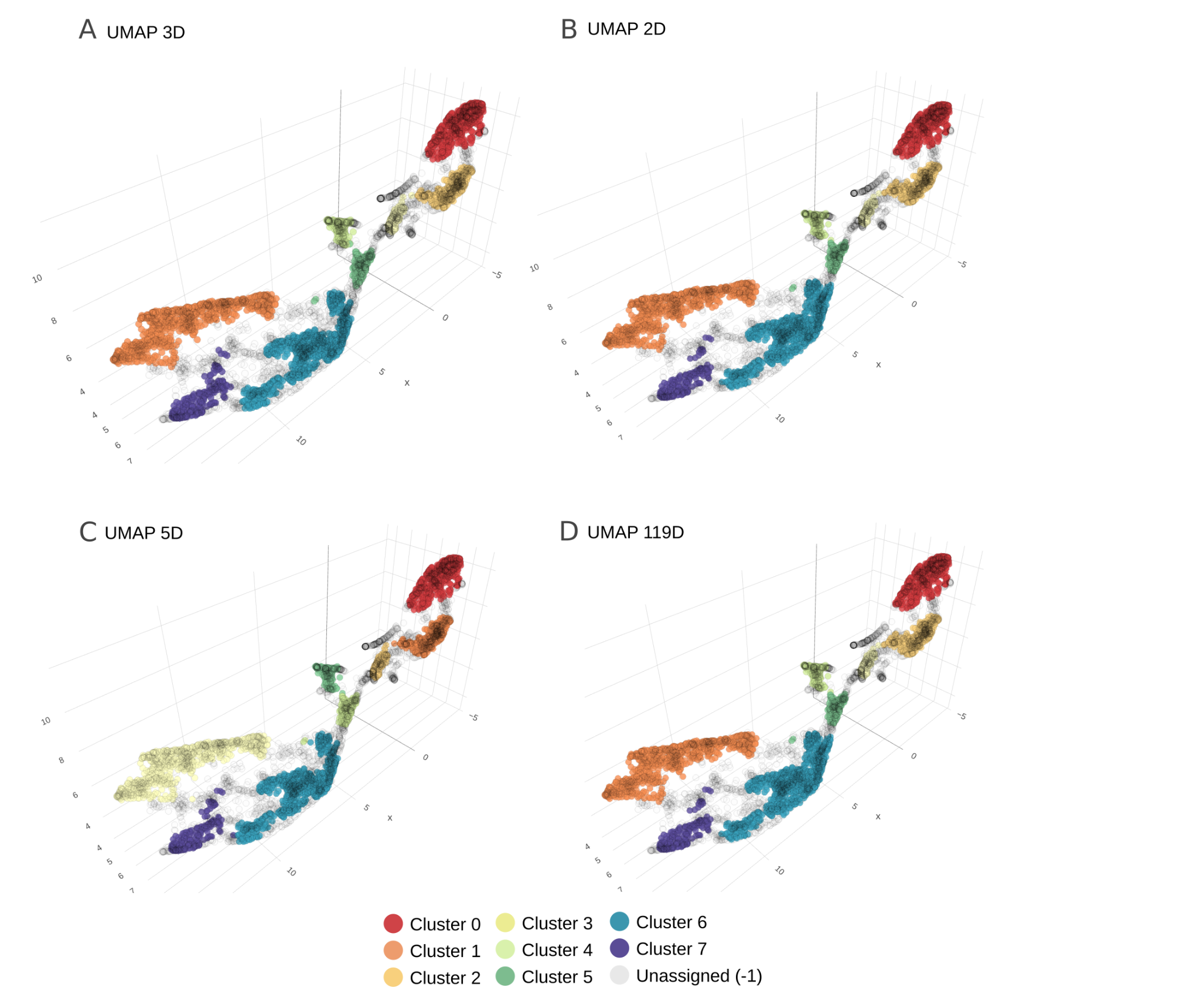 |
| --- |
| **Fig S2-Text-16:** **Unsupervised clustering of soundscapes recorded on the coral reefs of Bora-Bora (UMAP 3D view).**  UMAP visualization using HDBSCAN with the Leaf clustering method and a minimum cluster size of 100 samples. (A) Using 3D UMAPs. (B) Using 2D UMAPs. (C) Using 5D UMAPs. (D) Using 119D UMAPs. |

| 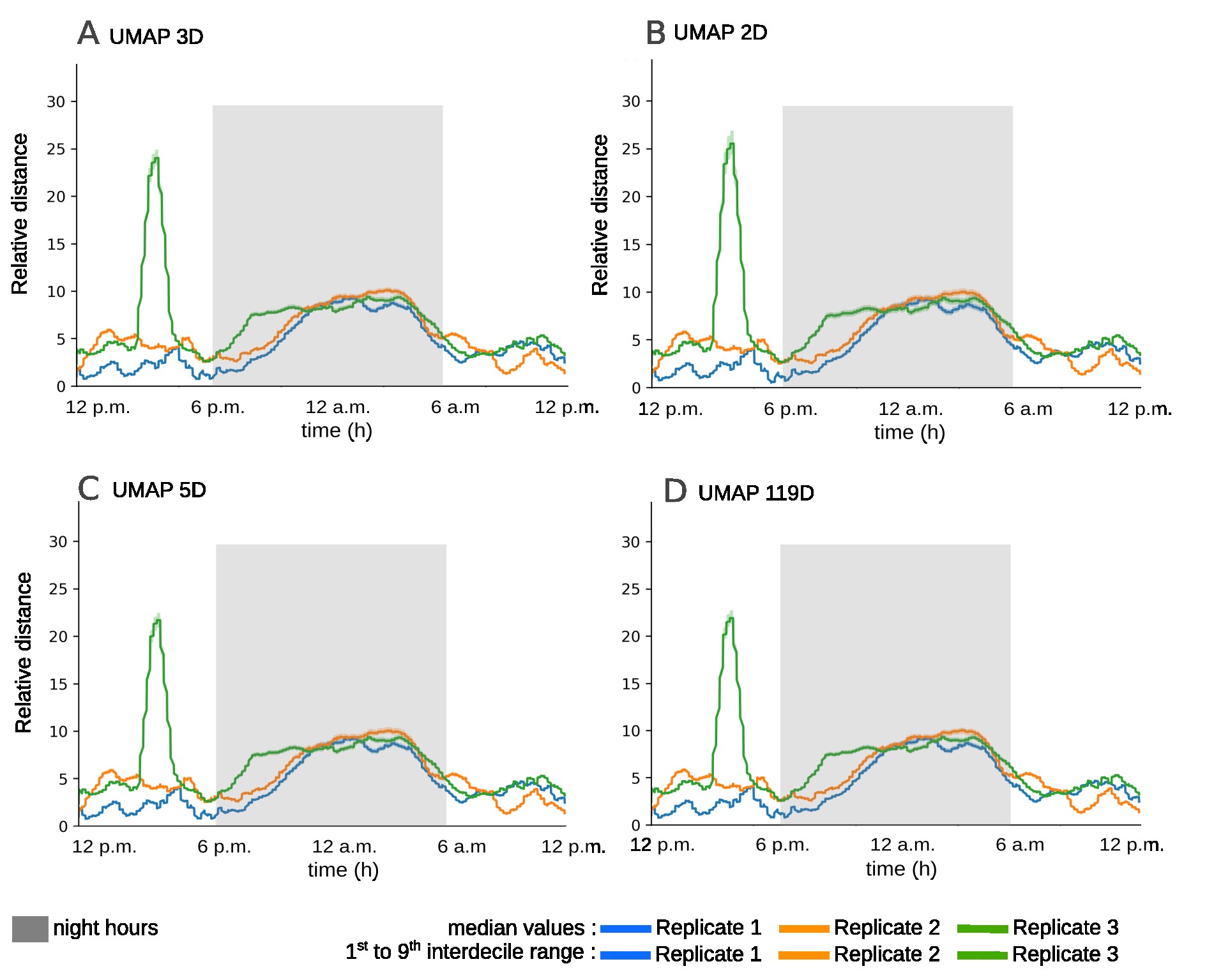 |
| --- |
| **Fig S2-Text-17: Time trajectories of the three replicates for the undisturbed site, considering different UMAPs dimensionalities.**  (A) Using 3D UMAPs. (B) Using 2D UMAPs. (C) Using 5D UMAPs. (D) Using 119D UMAPs. |

# References

1[. Han H, Li W, Wang J, Qin G, Qin X. Enhance explainability of manifold learning. Neurocomputing. 2022;500(C):877‑95.](https://www.zotero.org/google-docs/?broken=E5yspx)

2[. Wang Y, Huang H, Rudin C, Shaposhnik Y. Understanding How Dimension Reduction Tools Work: An Empirical Approach to Deciphering t-SNE, UMAP, TriMAP, and PaCMAP for Data Visualization. J. Mach. Learn. Res. 2021;22:201](https://www.zotero.org/google-docs/?broken=I19oz9)
