# Supplementary material for: Visualization and quantification of coral reef soundscapes using CoralSoundExplorer software
Source: PLoS Comput Biol. 2025 Apr 10;21(4):e1012050. doi: 10.1371/journal.pcbi.1012050 (PMC12017563; doi:10.1371/journal.pcbi.1012050)
Supplement: S3 Text — (DOCX) [file pcbi.1012050.s003.docx]

**S3 Text: Software installation procedure and instructions**

*CoralSoundExplorer* provides an efficient environment for working with acoustic data. Here we give a quick overview of how to install and use the software. Its source code and the dataset used for illustrative purposes in the present paper (Bora-Bora) are available online on GitHub<https://github.com/sound-scape-explorer/coral-sound-explorer>.

# Supported Platforms

*CoralSoundExplorer* is compatible with various operating systems capable of running Python 3 and Node.js. We have extensively tested the software on the following platforms:

1. **Windows**: Supported on Windows 10 and later versions.
2. **macOS**: Compatible with the latest M1 and M2 chips.
3. **Ubuntu**: Installable on Ubuntu 20.04 and newer.

# System Requirements

Prior to installation, ensure that your computer meets the following requirements:

- A minimum of 4 GB of RAM for optimal performance.
- A multi-core processor (2 cores or more) for efficient audio processing.
- About 15 GB of free disk space for installation of dependencies.
- Sufficient storage space for audio files and processed data.
- For optional accelerated audio processing, we advise using an NVIDIA GPU.

# Installation Instructions

To install *CoralSoundExplorer* on your machine, please refer to our detailed installation guides available here <https://sound-scape-explorer.github.io/docs/CSE/installation/>.

# Software Architecture

| 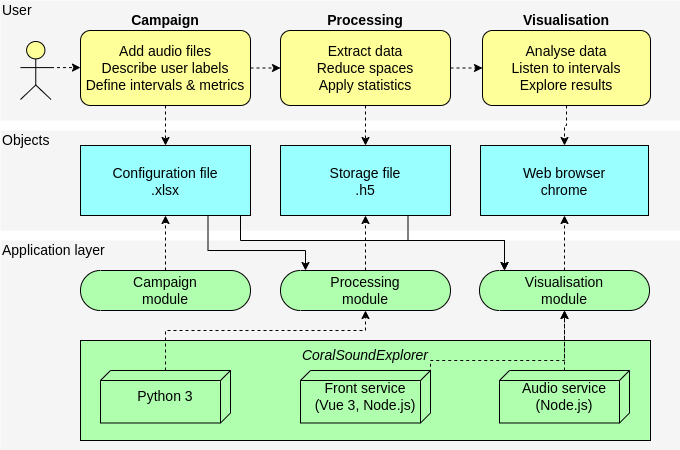 |
| --- |
| **Fig S3-Text-1:** **Architecture of *CoralSoundExplorer* software.**  The top panel describes the user actions. The second panel presents application objects the user interacts with. The bottom panel displays the application modules with underlying technologies. |

*CoralSoundExplorer* consists of three distinct modules: Campaign, Processing, and Visualization, tailored for campaign creation, settings customization, and data sharing (Fig S3-Text-1). These domains can be used independently, accommodating various usage scenarios.

## Campaign Module

Upon collecting field audio recordings, researchers can define configuration files and run new campaigns using *CoralSoundExplorer*. Detailed instructions can be found in thecampaign documentation<https://sound-scape-explorer.github.io/docs/CSE/modules/campaign/>.

## Processing Module

The Processing Module is the core of *CoralSoundExplorer* and is written in Python 3. It uses a series of algorithms to extract valuable metrics from audio recordings. The user must provide the system with a configuration file in .xlsx format, which guides the computation process by indicating the audio file paths and the necessary settings (default parameters are proposed). The generated data is stored in .h5 format, enabling seamless integration with the Visualization Module and facilitating data sharing between users.

The Processing Module is the most important component of *CoralSoundExplorer*, both in terms of size and performance requirements. Processing times for the Bora-Bora dataset taken as an illustrative example in this paper range from 40 minutes (on a GPU-accelerated standard desktop) to 3 hours (on a standard laptop with a CPU). For additional details, please consult theProcessing documentation <https://sound-scape-explorer.github.io/docs/CSE/modules/processing/>.

## Visualization Module

Implemented in TypeScript using Vue 3, the Visualization Module provides an intuitive and interactive graphical interface with an additional, independent Audio service. This web application operates locally, eliminating the need for an internet connection. A public instance is also availableonline at <https://sound-scape-explorer.github.io/docs/CSE/extras/visualisation-online>, enabling basic exploration without any installation. Users can input h5 storage files generated by the Processing Module (including files they have not generated themselves, but have shared with other users). The Visualization Module offers a comprehensive set of features for visualizing and listening to processed results, empowering researchers to gain insights and make data-driven decisions.

The Audio service is a lightweight web server designed to seamlessly integrate with the Front service (web application). It enables real-time playback and spectrum analysis of audio files.

For a detailed guide on using the Visualization Module, please refer to our documentation <https://sound-scape-explorer.github.io/docs/CSE/modules/visualisation/>.

# Future development

To facilitate bug fixes and the addition of new features, we encourage users and researchers to share their feedback and suggestions by creatingnew GitHub issues. Please read our contribution guidelines <https://sound-scape-explorer.github.io/contributing>. Your input is invaluable for the continued improvement of *CoralSoundExplorer*.
