# Supplementary material for: Visualization and quantification of coral reef soundscapes using CoralSoundExplorer software
Source: PLoS Comput Biol. 2025 Apr 10;21(4):e1012050. doi: 10.1371/journal.pcbi.1012050 (PMC12017563; doi:10.1371/journal.pcbi.1012050)
Supplement: Attachment — Submitted filename: Response to comments_PCOMPBIOL-D-24-00575.docx [file pcbi.1012050.s009.docx]

Dear Frédéric E. Theunissen,

We are pleased to submit our revised manuscript entitled “**Visualization and quantification of coral reef soundscapes using CoralSoundExplorer software**” by Minier et al. (**PCOMPBIOL-D-24-00575**). Your comments and suggestions have enabled us to improve this article considerably, involving numerous changes to both content and organization. We would like to thank you very much for taking the time to work on our article and hope that you will be satisfied with this new version. Of course, we remain at your disposal should you wish to make any further changes.

Briefly, here’s what we have done. The part detailing the method has been placed in “S1 Text: Detailed Methodology of *CoralSoundExplorer*”. Your questions about the effects of certain software options and parameters on the results have been addressed in a new section entitled: “*CoralSoundExplorer software*: A parametric study with the Bora-Bora dataset”. Looking at the results produced by the software on the analysis of the Bora-Bora dataset, we now address the questions of the effect of the initial acoustic projection methods, the effect of the dimension reduction method and the effect of the dimensionality of the UMAP projection. We have also moved to this section the study of the effect of averaging UMAPs, which we have completed by quantifying this effect on the contraction of distances between points. In the main manuscript, the results are discussed in the text only. Due to their number, we have chosen to place the corresponding figures and their raw descriptions in the supplementary material “S2 Text: Parametric study of *CoralSoundExplorer*”.

We understand the suggestion to publish this article as a “software article”. However, this article is much more than a simple presentation of a new software. In fact, the article proposes and evaluates the relevance of new analysis tools for monitoring coral reef soundscapes (e.g. “Temporal trajectories in UMAP”) and analyzing similarities or dissimilarities between them (e.g. “Silhouette index matrices made from several UMAPs averaging”). This article also provides new ecological results on coral reef soundscapes (by presenting, for example, the phenology of coral reef soundscapes and their differentiation according to recording locations). The practical consequence of these different aspects covered in the article is that it cannot fulfill the submission guidelines for a “software article”. The number of words imposed (3500) is too limited for the amount of information provided (we have in fact placed the software installation instructions in the supplementary materials: “Text S3: Software installation procedure and instructions”). If it's alright with you, we'd much prefer to keep this article as a classic research or methods paper, with no particular constraints on its length that would considerably diminish the scope and interest of our work.

However, we are aware that some readers of your journal might be interested in an article focusing solely on the didactic presentation of our software. We therefore have a proposal. As mentioned in the conclusion of the present article, we are currently finalizing a more general software solution than CoralReefExplorer, dedicated to the study of any marine or aerial soundscape. This new tool features a similar unsupervised framework and interface to CoralSoundExplorer, but has broader applicability to a variety of soundscapes and includes additional analysis features. If you are interested, we can prepare a second article, this time for the ‘software’ section of your journal and therefore considerably shorter than the present article, which will introduce this generalist software to your readers. Please let us know what you think.

Please find below a point-by-point response to reviewers’ comments. Changes to the revised manuscript are shown in yellow. We hope you find our revision relevant for publication and thank you warmly for your time and consideration.

Kind regards,

Jérémy Rouch, on behalf of co-authors.

**Reviewer #1:**

Here are some of the strengths of the paper. First, it is clear that this will be a useful product for this community and I count on you to advertise your software and this paper. Second, I like that in the paper you stress the significance from an ecological perspective of monitoring our environments and corals in the ocean in particular. This is well described and well referenced in your introduction. Third, although your paper is long, it reads very well. The description of the methods is very clear. There are a few places where you are a bit vague (see minor comments) but you will be able to fix that. Finally, I also like you use the Bora-bora data set to illustrate the three types of analyses that are greatly facilitated by *CoralSoundExplorer*: supervised classification, clustering, temporal trajectories. As a Software Article, it has very few problems. I will, however, bring to your attention some issues that I find are relatively major. They have less to do with the software per se and more with thinking about how to maximize the biological impact of these measurements. I hope you can address most of these in your revision. You will see that some of the points that I bring to your attention were also raised by the reviewer (I read the paper without reading his review)**.**

Thanks for the positive feedback and the valuable comments. We addressed them to improve our manuscript.

*More major comments.*

1. The dominant effect appears to be the recording site. Although this provides a clear, illustration that the methods “works”, it also raises the issue that the distance between sound features are dominated by “average” acoustics, which in turn are dominated by environmental factors over biological sound sources. Taking into consideration that I am somewhat of an outsider to this field, it seems that using microphone arrays and performing some sort of sound source separation before feeding the sounds to the analysis pipeline would result in a much richer analysis. This should at least be discussed and, maybe, something to add to the software?

In part : “III. *CoralSoundExplorer* software: Results obtained with the Bora-Bora dataset - Conclusion on the Bora-Bora case study”, we've added the following text to emphasize the dominance of the effect of acoustics over isolated sounds. We also discuss the possibility of using either spatial (microphone array) or computational filtering approaches to better highlight certain biological sounds. [L 644-668]

“[...]unsupervised classification identified groups of sound samples that were not distinguished by predefined labels, but differed in terms of shrimp sound quantities, fish sound quantities and types, broadband background noise levels and frequency balances, and the presence of boat noises. However, the analysis carried out here on the Bora-Bora data set did not reveal groups characterized solely by fish sounds. Numerous species of sonorous fish are probably present and active at these sites, and identifying these groups would probably enable a more in-depth ecological study. The absence of distinct groups of different fish sounds can be explained by three possibilities, not necessarily mutually exclusive. Firstly, the integration time chosen was 15 seconds, which may be a little long compared with the usual duration of fish sounds, the usual short periods of fish vocal activity and the variety of species vocalizing almost simultaneously. These isolated sounds could also be acoustically insignificant compared to the overall background noise that dominates the recordings in terms of consistency and level. A way to compensate for this problem of confounded signals in the background noise would be to use a preprocessing step of isolation of punctual acoustic events, such as the use of hydrophone arrays or numerical denoising methods. Secondly, the initial acoustic feature extractor may not be sufficiently specialized in fish sounds and CoralSoundExplorer could benefit from another CNN more dedicated to biological sounds. Finally, the overall acoustic differences between sound samples according to site, replicate, time of day or presence of boat and rain are greater than between the presence or absence of different fish species. Considering the entire recording campaign, the UMAPs used prior to the clustering process may have contrasted distances more according to site/replicate/period/boat/rain characteristics than according to the presence or absence of different fish species. One possibility to potentially increase the identification of fish sounds clusters would have been to work separately by site and period of day, so these categories might not have dominated the soundscape differences.

”

2. To interpret the unsupervised clustering, you propose to use the audio monitoring of samples in each group. But the only interpretation you report is boat vs less boat in the boat site. This is a bit unsatisfying. I am guessing that if you had a pipeline that included sound source separation, your unsupervised clustering would be more interesting.

We have added details in the interpretation of Fig. 12-C&D on the results of unsupervised clustering with HDBSCAN's “Leaf” method. We have added interpretations, focusing more on biotic sounds (see below). The possibility of using a source separation pipeline is mentioned in the reply to your previous comment. [L 535-560]

“Automatic clustering using the HDBSCAN with Leaf method identifies eight clusters (Fig. 12C). Clusters 0, 1, and 2 exclusively contain recordings from the boat site. Cluster 0 primarily consists of nighttime recordings (93.68 %) with constant shrimp sounds and isolated fish sounds characteristic of the boat site at night. Clusters 1 and 2 share daytime recordings (respectively 97.86 % and 90.44 % – Fig. 12D & S4 Table). After an audio monitoring of these sounds with the CoralSoundExplorer audio server, we observed that these two clusters differ in the nature of the soundscape. Sound signals contained in cluster 2 exhibit clear emergences of motorboat sounds with distinct isolated frequency components. Cluster 1 contains more broadband background noise. Both cluster sound samples may also be composed of various fish sounds. Sounds from both tourist and undisturbed sites are represented in cluster 3 (64.95 % composed of undisturbed site recordings and 35.04 % of tourist site recordings) and cluster 6 (44.58 % composed of undisturbed site recordings and 55.41% of tourist site recordings), which are characterized by recordings common to coral reefs. Cluster 3 primarily consists of nocturnal recordings, very rich in impulsive sounds mostly due to shrimp activity with a low background noise level. Cluster 6 (Fig. 12C) consists of daytime recordings, composed of fewer impulsive sounds but with a higher presence of low frequencies. Both cluster sound samples may be rich in various fish sounds, which is likely an indicator of the presence of healthy corals at the tourist site. Clusters 4 and 5 are exclusively composed from recordings of replicate 3 of the tourist site, respectively nighttime and daytime at 94.68 % and 90.54 %. Both clusters are noisy, with the distinction that cluster 4 exhibits very loud shrimp clicks that are not present in cluster 5. Cluster 7 is composed of undisturbed site recordings during the first two days. These recordings are quieter than the others, with little background noise and few clicks. Clusters 4, 5, and 7, as well as the others, contain fish sounds. An equal number of recordings from the three sites were not assigned to a specific cluster (unassigned (-1), 36.06 % from the undisturbed site, 29.96 % from the boat site, and 33.97 % from the tourist site).”

3. I have some worries about the use of a UMAP metric to look at temporal trajectories – as you know a non-linear embedding will distort distances – this is clearly an analysis that needs to be done with a systematic changes of some hyperparameters (see 5 below) and with a plot that provides error bars from both randomness (your 100 UMAP realizations) and sampling through hyperparameter space.

In both the manuscript and the software, we have now included a measure of dispersion in the graphical display of relative distances (the interdecile between the first and ninth decile). The dispersions observed are generally very low, showing good stability of this metric from one UMAP to the next. The random nature of UMAPs therefore has little influence on this metric. The analysis of these graphs was also the subject of a systematic study during the parametric study of the software to see the influence of several hyperparameters on this metric.

4. Your interpretation of the results for these temporal trajectories are also very descriptive with a minimum of biological interpretation. I believe this is a combination of the issues in 1 and 3. Along this point, you state in the discussion that “Dynamic variations in soundscapes over a 24-hour cycle are also highly informative.” I think that “highly informative” is a bit of an exaggeration given the observations you made here. I agree that such an analysis could be informative but here you mostly have illustrated the method.

We modified the sentence and removed “highly informative” (L669 - 686).

5. Detailed Methodology should be a Method Section or a Supplementary section.

As suggested, we moved this section into a Supplementary Section S1-Text.

6. In the methods, I would add the equation for the mel-frequency bands that you used. Also, what is the justification for using something that was pretrained from 125 to 7500 Hz if you then apply it to a 70 to 2000 Hz. Maybe you would get better results by mapping it to fewer bands between 125 and 2000 Hz but actually matching? Or if there is some physical reason for doing that transposition because of water vs air speed of sound, you should probably justify it and use the actual physical factor.

As suggested, we added a reference to the mel frequency scale equation (Douglas’ formula). [S1-Text; L 65]

As the VGGish network was made to analyse sound’s spectrogram data structures but without being specialized for fish or underwater sounds (because probably poorly trained on these kinds of sounds), we firstly preferred to use spectrograms as VGGish inputs that matched the fish sounds frequency band instead of the spectrograms as those used for the VGGish training. However, in the new section “CoralSoundExplorer software: A parametric study with the Bora-Bora dataset - Choice of the initial acoustic projection space”, we addressed the question of the effect of the initial acoustic projection space. We compared the VGGish 70-2000 Hz embedding and the VGGish 125-7500 Hz embedding. A spectrogram on fewer frequency bands before using the VGGish network is unfortunately not possible, since the VGGish network requires exactly 64 of mel. However, as your comment overlaps with that of the other reviewer on the justification for using the VGGish network for a frequency band different from that for which it was designed, we included the VGGish 125-7500 Hz embedding (the original band) in this study. The results of this parametric study do not show a clear superiority of an embedding over the other except a more distinct effect of the rain event on the temporal trajectories for the undisturbed site when the VGGish 70-2000 Hz embedding is used. [L 705-745; S2-Text L3-40]

7. Related to 6, what kind of results would you get if you fed the spectrogram (or mel-spectrogram) directly to UMAP. A great improvement obtained by using VGGish would further sell your approach.

We thank the referee for this very interesting suggestion. In the section “*CoralSoundExplorer* software: A parametric study with the Bora-Bora dataset”, when addressing the question of the effect of the initial acoustic projection space (see answer to 6) we also included the mel-spectrogram and the mel-spectrum as initial embeddings. The comparison of the results given by *CoralSoundExplorer* have not shown clear differences when using the VGGish 70-2000 Hz embedding or the mel-spectrogram, even for the rain event that had a similar relative effect on the temporal trajectories (the mel-spectrum showed a less relative effect of this rain event on the temporal trajectories, as for the VGGish 125-7500 Hz embedding). In the manuscript we made the following hypothesis to explain this equivalence of results : “This result may seem surprising, as VGGish was designed to capture more complex sound structures than spectrum and spectrogram. However, the VGGish network was trained on a dataset that likely contained very few, if any, coral reef sounds. Consequently, it may rely primarily on basic spectrogram features to project these soundscape samples.” [L 864-867]

8. I found the “conclusion” section “Main achievements and limitations” a bit redundant and the limitations that you state are mostly of computational nature (and thus quite minor). A more candid review of the more critical limitations of the entire procedure would be appropriate.

We have extended our conclusion to include a critique of the method. We insisted on the main difficulties induced by working with unsupervised approaches: difficulties to have “ground truth”, the “turn button” approach, explaining unsupervised clusters, and giving sense to the processing (i.e. understanding unsupervised features). [L 922-960]

“Limitations of CoralSoundExplorer

The results of the parametric analyses conducted here (for instance on the number of UMAP dimensions or the initial acoustic space projection method) are only really valid for the dataset used and unsupervised learning methods are sometimes difficult to handle and still require a non-naive point of view when interpreting results and adjusting parameters. Although coral reef soundscapes all share common characteristics, it is possible that for datasets larger and more complex, the values of 3 dimensions and 100 UMAPs are not suitable for a good representation of data organization and convergence of these representations. Also, the question of the UMAP, spectrogram representation or HDBSCAN hyper parameters have not been investigated in this study while they may have an influence on the results. This question of parameters or hyperparameter study is one of the main difficulties when working with unsupervised learning methods such as proposed by CoralSoundExplorer. First because it could be confusing when working with a “blind turn-buttons” approach especially when multiple parameters are involved and could lead to cross effects. Secondly, because the unsupervised approaches often have no exact “ground truth” to rely on, it’s difficult to completely confirm the validity of the results obtained. This difficulty of dealing with unsupervised learning techniques is particularly well illustrated by the identification of unsupervised clusters. In the case of the CoralSoundExplorer we chose to work with HDBSCAN algorithm. As other clustering algorithms, one of the main difficulties arises from the fact that it can give different results depending on its parametrization. Although here, the two different parameters of the final clustering method (“EOM” or “Leaf”) lead to results consistent with the spatio-temporal analysis of the recordings, it is possible that with a different parameterization, a finer or coarser clustering would have emerged and also made sense, or on the contrary, would have produced meaningless subgroup clusters. To evaluate the consistency of the clusters, it is always advisable to go back to listening to the sounds or visualizing their spectrogram. However, even if this listening possibility can help the operator to validate the clustering results, the acoustic characteristics used by the software to distinguish or not sound samples remain hidden, even more with the use of a CNN embedding.

The parametric study also gave a good estimation of the efficiency of each workflow step. The choice of the UMAP processes seems to be an important element for the unsupervised analysis developed for CoralSoundExplorer. The VGGish network can effectively give important insight about similarities and differences between coral reef soundscapes and their organizations, but it appears that the mel-spectrogram could perform almost identically as initial acoustic embedding. A network more dedicated to biological sounds detection in natural recordings, such as birdNet [83] or Perch [84], could provide a more suitable embedding for natural soundscapes study [85], more optimized for animal vocalisations, and thus give more information on the composition of soundscapes.”

“However, unsupervised learning methods are sometimes difficult to handle and still require a non-naive point of view when interpreting results and adjusting parameters. For the Bora-Bora case study discussed here, we have presented a limited set of interpretations focusing mainly on the raw results provided by *CoralSoundExplorer* without going too far into ecological considerations. However, further analysis could be carried out to explore in depth the relationships with biodiversity, reef health and anthropogenic disturbance.”

*Minor Comments:*

1. Author Summary l47-49. I know that you are providing a high-level overview but I think that you could be more precise in these sentences. As written, it sounds like something that one would read in an advertisement

We have revised the author’s summary to add more details about the proposed method and to adopt a less “advertising” style. [L 45-65]

“Passive acoustic monitoring is an increasingly popular method for monitoring ecosystems, but analyzing the data generated by this approach remains complex. In this paper, we present CoralSoundExplorer (https://sound-scape-explorer.github.io/docs/CSE/), a tool for analyzing large sets of sound recordings. The tool is open source and has been designed to be easy to use, even by non-specialists, thanks to a graphical interface. This graphical interface is also available in an online version to allow users to visualize previously processed data without needing to install the software (https://sound-scape-explorer.github.io/coral-sound-explorer/). The CoralSoundExplorer software transforms recordings of coral reef soundscapes into visual representations using UMAP embeddings coupled with tools for quantifying phenomena. By projecting sound samples into acoustic spaces, CoralSoundExplorer enables the observer to grasp the characteristics of the soundscapes, their differences and similarities and their organization on different temporal scales. These acoustic spaces and their temporal dynamics can be quantified, for example to account for the speed at which soundscapes change over time or to identify clusters of sound samples on the basis of their acoustic similarities. We use an example of the reef soundscapes of the island of Bora-Bora to present and illustrate the functionalities of our software, confirm previous findings, provide new insights, and demonstrate the applicability of the software for the analysis of large datasets. Based on the Bora-Bora dataset, we also provide a parametric study of CoralSoundExplorer to show the effects of its different settings on the analysis results. Detailed instructions for installing and using the software are also provided in the supplementary materials.”

2. Intro l100. Related to point 1, you could very briefly state what terrestrial indices are attempting to measure (and why it might not be good for aquatic environments).

We have added a few details about terrestrial acoustic indices and their limits. Especially for applications in the marine environment. [L 106-123]

“Soundscapes are commonly described using acoustic indices [40]. In terrestrial environments, a chosen set of acoustical indices can be used to perform statistical data analysis in order to infer ecological results. Correlation studies and dimension reduction techniques can be applied to sets of acoustic indices to explore differences between soundscapes and their evolution. From a set of indices, clustering methods can be used to perform semi and unsupervised analysis using machine learning tools. Such approaches have already been applied to coral reef soundscapes, using for example the Acoustic Complexity Index (ACI) [41], Acoustic Entropy (H) [42], Acoustic Diversity Index (ADI) [43]. The ACI measures the average temporal variations in frequency band levels [44] and is assumed to correlate with the ratio of biotic to anthropogenic sounds. ADI measures the Shannon entropy of level values over several frequency bands, supposed to be related to the diversity of sound sources over the corresponding frequency band(s) [45]. Acoustic entropy measures the Shannon entropy of the amplitude envelope and/or spectra of sound signals [12] and is related to the temporal and spectral diversity of sounds. However, a growing body of experimental evidence suggests that terrestrial acoustic indices are not ideal for characterizing underwater soundscapes, and may even perform poorly for specific terrestrial soundscapes [5,30,46], as differences in sound propagation phenomena, source types, spectral characteristics and temporal dynamics lead to ecosystem-specific organization and composition of soundscapes.”

3. L183. “However, such tourism access..” . You don’t need “however” here since you are not negating the previous statement.

We have changed the sentence (L 209-210).

“This tourism success comes with a price, as it generates significant maritime traffic, inevitably accompanied by noise pollution caused by the intensive use of internal combustion engine-powered boats.”

4. L240. I would state that VGGish is trained on the sound of annotated YouTube videos.

We have specified it. (L 280-283)

“[..]the mel-spectrograms described above [...] are fed into a Convolutional Neural Network (CNN) pre-trained on a large YouTube’s audio-video dataset named VGGish [73]”

5. As mentioned above, your treatment of the hyper-parameter fit for the UMAP is quite comprehensive. Nonetheless, it would have also been interesting to see results of actual clustering and time-courses for different dimensions. You can see that I am making this point both as more major (because of the sensitivity for the temporal trajectories) and minor here (because you did a nice job exploring the space of hyperparameters for UMAP)

In the section “CoralSoundExplorer software: A parametric study with the Bora-Bora dataset - Choice of UMAPs parameters”, we moved the part exploring the UMAP parameter. We improved this part by adding the effects of the UMAP dimensionality and averaging on the results given by the software (silhouette indices, unsupervised clustering and temporal trajectories). [L 815-822; S2-Text L 162-174]

“Considering the RTA and the DLP metrics, only the 2D UMAPs is less informative, thus any of the 3 highest dimensionalities (3D, 5D or 119D) considered should be preferred to compute the 100 UMAPs. Considering either the silhouette matrices obtained, the unsupervised clustering results or the soundscapes time trajectories, no effect of the UMPAs dimensionality was observed. Thus, the 3D UMAPs reduction method was retained to work with the Bora-Bora dataset because this low dimensionality is sufficient to keep the most possible of the data information, considering the RTA and DLP, and is consistent with the 3D visual plot offered by CoralSoundExplorer.”

6. In the legend of Fig 6, I would specify that the unsupervised cluster with label -1 is what HDBSCAN has classified as “noise”, or more precisely as having low probability of belonging to any of the clusters.

We have changed the legend of Fig. 6. [L 368]

“Fig 6: Analysis offered by CoralSoundExplorer: Unsupervised sound clustering (here 3D UMAP view).

Graphical display possibilities: Color points (sounds) according to the values of unsupervised clusters id (cluster label -1 is related to unclassified sound samples), Contingency matrix (i.e., percentage of each category of one categorical variable within each category of another categorical variable). Export possibilities: UMAP/PCA plots (.png, .svg), contingency matrix (.csv) and its plot (.png, .svg).”

**Reviewer #2:**

The paper describes a novel software tool that uses a pre-trained VGGish network model as a feature extractor and UMAP to generate low-dimensional visualisations. UMAP is also used to compute a distance matrix for subsequent analyses - this is achieved by averaging over multiple UMAP runs. The results presented for the Bora-Bora dataset (e.g. meaningful, visually discernible clusters, state space trajectories, and contingency tables presented on an interactive interface) demonstrate the usefulness of the tool for soundscape analysis.

In fact, the relevance of the tool is broader than it might first appear: apart from a few (editable) parameter choices, there doesn't seem to be anything that would make it exclusive to coral reef environments. The generalisability to other datasets (of either same or different biomes), however, has not been demonstrated. As an example, the choice of the number of UMAP replicates was based on convergence analysis (Figure 17), but the results may be dataset-specific (as acknowledged by the authors on page 46). Applying the method to different datasets would certainly be helpful - though not a requirement. As an alternative, a better understanding of the method, in particular the distance matrix averaging procedure, could help ensure robust performance across diverse datasets.

On this topic, a major point is raised below. In addition, a number of minor comments are also offered.

We thank Reviewer #1 for the positive feedback and the comments that helped us to improve the manuscript.

*Major comments*

Stochastic neighbourhood embedding methods like UMAP create low-dimensional representations that aim to preserve the distance structure present in the high-dimensional data, but this inevitably introduces distortions due to approximations (e.g. limited neighbourhood size) and stochasticity. The latter, in particular, affects reproducibility. To address this issue, the authors propose running multiple replicates of UMAP and using the average distance matrix for downstream tasks. However, this approach raises questions.

1) As is generally the case with stochastic estimation, averaging over multiple runs should yield a better approximation of the distance matrix. However, the distance matrix in the original VGGish embedding space is accessible. In fact, an estimate of it (based on neighbourhoods of limited size) is used as input to compute the UMAP embeddings. Why going through the trouble of averaging over 100 replicates of UMAP estimates of the distance matrix when the original one is accessible?

The answer to this question seems to have to do with the curse of dimensionality (and the related distance concentration problem): as the number of dimensions increases, the volume of the space increases exponentially. This leads to data points being sparse and spread out, which in turn makes distances between points less meaningful (see "On the Surprising Behavior of Distance Metrics in High Dimensional Space" by Aggarwal et al.). That poses problems for clustering, as confirmed by the authors with the Hopkins statistics for 3D (single run UMAP) vs 119D (original VGGish embedding) spaces [line 807]. Reducing dimensionality is thus a good idea. In fact, it is common practice (eg, recommended in UMAP python package documentation, <https://umap-learn.readthedocs.io/en/latest/clustering.html>).

This question is indeed linked to the curse of dimensionality. To answer it more fully, in the new section “CoralSoundExplorer software: A parametric study with the Bora-Bora dataset”, we have included a parametric study of the data reduction method. We compared the effect of using UMAP, PCA or directly integrating VGGish. The study shows that the use of UMAP has the advantage of increasing the contrast of distances between points, and thus improving the contrast between clusters of points. [L 756-795; S2-Text L 41-80]

“Choice of the dimension reduction process

The method of dimension reduction applied to the initial acoustic projection space can also be questioned. By default, CoralSoundExplorer uses UMAP, a non-deterministic approach. As previously mentioned, the distance matrix between sound samples required for the HDBSCAN algorithm and the silhouette matrix is computed by averaging results from multiple UMAP iterations. Here we chose to set the number of iterations to 100. Alternatively, PCA can be employed for 3D visualization and/or for the distance matrix. Another option is to calculate the distance matrix directly from the initial acoustic projection space. Because PCA and the direct VGGish embedding process are deterministic, they eliminate the need of an averaged distance matrix, consequently one realisation of them is enough to have the distance matrix.

Comparing the 3D UMAP, 3D PCA, and direct VGGish 70–2000 Hz approaches, the silhouette values for the site/replicate/period label show for the 3D UMAP more contrasted values, more marked differences between the categories of this label. 3D PCA and direct VGGish embedding are more similar within each other. They also both reveal more similarities between a few categories of this label. The similarity between PCA and direct VGGish embedding is not surprising, as 3D PCA performs a linear transformation of the VGGish features, and the three most significant dimensions explain a not so small proportion of variance: approximately 45%. The number of unsupervised clusters identified with 3D PCA and without dimension reduction is significantly reduced, yielding only 3 and 2 clusters respectively while 3D UMAPs produced 8 clusters. However, the contingency matrices comparing these clusters to the composite site/replicate/period predefined label demonstrate logical results. The clusters observed with the 3D UMAPs process are subdivisions of the clusters obtained by the other approaches. The temporal dynamics of soundscapes at the undisturbed site over the three recorded days show qualitatively consistent results across the three approaches. The first two days exhibit similar dynamics, while the third day diverges during the rain event. However, the 3D UMAP approach highlights a greater relative amplitude of this deviation compared to the other methods. The superiority of the UMAP reduction process to distinguish between the site/replicate/period categories and to highlight an isolated soundscape deviation drove our decision to work with the UMAP reduction process. This superiority of using the UMAP on the initial acoustic space (VGGish embedding) is not so surprising. The initial acoustic space is high dimensional and the data benefit from this situation according to the concept of “the blessing of dimensionality” which stands that in high dimensional spaces data are more linearly separable [76]. However, this effect is balanced by the “curse of dimensionality” [77] which stands that high dimensional data are sparse and that this leads to difficulties in finding potential separation between data. To deal with the “curse of dimensionality”, a dimension reduction process is often used but the PCA is not based on the notions of distances and separability while the UMAP is based on them by trying to keep the distance relations and is known to increase the clustering effect [78].“

That brings us to the second question:

2) Doesn't the proposed procedure (ie, averaging the distance matrix over multiple UMAP runs) reintroduce the problems faced in high dimensional spaces (ie, render pairwise distances less informative)? Or is it really possible to reap the benefits of a more faithful approximation of the original distance structure while still avoiding the curse of dimensionality?

To properly address this point, a number of methods for assessing distance concentration exist. One possible analysis in the spirit of figure 17 is to measure the coefficient of variation of distances (similarly, IQR over median, and contrast of distances) of D(n), n in N = (1, 100). The plotted values are expected to be stable (good for the proposed method) or decay (not so good). The baseline for comparison should be the same metrics (CV, IQR/median, and contrast of distances) applied to the original VGGish space.

We have followed your recommendation by incorporating a stability study of the IQR/median (or IQM in the manuscript) metric as a function of the number of UMAPs performed to calculate the average distance matrix. This addition allowed us to confirm the deconcentration of distances thanks to the UMAP and showed the stability of this concentration with the number of UMAPs.

Thus we added the following paragraph in the section “CoralSoundExplorer software: A parametric study with the Bora-Bora dataset - Choice of UMAPs parameters” [L 866-884; S2-Text L 109-120]:

“Besides the convergence of the distance matrices, we wanted to know if both this dimensionality and the averaging process has an impact on the relative repartition of the distances within the average distance matrices, i.e. if these parameters influence the effect of the UMAP on the reduction of the “curse of dimensionality”. To address this question, we calculated a concentration metric of the pairwise distances. For each of the n=100 successive mean distance matrices previously obtained, we calculated the interquartile range of the pairwise distances divided by their median (IQM). The idea was to check if the UMAP process effectively increases the pairwise distance contrast and if the averaging process of distance matrices obtained from independent UMAP does not decrease this effect if it exists.

For all UMAPs dimensionalities considered, after a small increase, the IQM values are rapidly stable according to the number of successive UMAPs computed for the mean distance matrix. Compared to the distances in the VGGish embedding, the UMAPs process double the IQM even after only one realisation. This proves that the UMAP process is effectively reducing the sparsity of the projected samples and that the averaging process of distance matrices does not reduce the distance contrast. The 119D and 5D UMAP transforms led to a smaller value of this contrast metric than 3D and 2D UMAP transforms, thus a very low number of dimensions for the UMAPs transform process increased the separation effect between points.”

*Minor comments*

127: The interface is described as "easy-to-grasp," but its usability has not been explicitly evaluated (e.g., with a user study). Instead of using this term, it could be stated that the interface was designed with the objective of being intuitive and user-friendly. This clarifies that ease of use is a design goal, although it hasn't been formally evaluated.

We have adapted this sentence (L 140-143)

“Our other aim is to provide the user with an intuitive and user-friendly tool, where Sethi et al. (2020) only offered a workflow indicating how to use a set of existing or specially developed programming language packages.”

128: "AI deep integration" is not a technical term I'm familiar with. Perhaps what is meant is that the tool integrates a (large, pre-trained) deep learning model into an interactive system.

This sentence has been changed (L146-149):

“Here we introduce a new, powerful analytical tool with a graphical interface designed with the objective of being intuitive and user-friendly, named CoralSoundExplorer. It uses machine learning techniques to analyze, describe, and quantify underwater soundscapes.”

130: Typo: convolutional instead of convoluted. It might be relevant to mention that the CNN has been pre-trained on a large audio dataset.

This sentence has been changed [148-152]:

“The CoralSoundExplorer workflow uses a initial acoustic embedding that can be either obtained from a Convolutional Neural Network (CNN), a mel-spectrogram or a mel-spectrum, followed by a Uniform Manifold Approximation and Projection (UMAP, [57]) to replicate the geometric relationships of the original high-dimensional space in lower dimensions.”

132: Suggested rephrasing: instead of "efficiently capture...data": something along the lines of "replicate in lower dimensions the same geometric relations present in the original, high dimensional space"

The corresponding sentence has been changed according to your comment (see our answer to your previous comment).

150: It is not clear what is meant by "measure the metrics"

As the paper has been reorganized, the sentence introducing part IV has been removed. The previous part IV has been moved to a supplementary section.

The equivalent text in the new version of the manuscript is as follows:

“ In section IV, we present a parametric study of the software. Using the Bora-Bora dataset, we explore the effects of the initial acoustic embedding and those of the dimension reduction process and its parameters. Section V examines the main achievements and limitations of CoralSoundExplorer, and presents our recommendations for exploring reef recordings with our software. We have added three supplementary sections to the document. The first complements Section II by providing detailed information on the software workflow and the proposed analysis, thus offering a more in-depth explanation of the methodology. The second completes section IV by providing figures and raw descriptions of the parametric study results. The third supplementary section describes the software structure and installation procedure.”

214: The frequency band of interest and sample length don't seem to match that expected by VGGish (125-7500 Hz, 0.96 s, <https://github.com/tensorflow/models/blob/master/research/audioset/vggish/README.md>). Can the authors please clarify this mismatch?

The explanation of the frequency band mismatch is discussed in the new manuscript: “CoralSoundExplorer software: A parametric study with the Bora-Bora dataset-Choice of the initial acoustic projection space”. The 70-2000 Hz band was used to match fish vocalizations, but we also tested the possibility of using the original VGGish frequency band (125-7500Hz).

“Choice of the initial acoustic projection space

Regarding the choice of initial acoustic projection space, we compare the effects of the three processes available in CoralSoundExplorer on the Bora-Bora dataset: the mel-spectrum, the mel-spectrogram, and the VGGish embedding. For the VGGish setup, we evaluated two frequency bands: 70–2000 Hz and 125–7500 Hz. The 70–2000 Hz was also used for the mel-spectrum and the mel-spectrogram computation. This frequency band corresponds to the expected range of fish sounds and the 64 mel band filters used by these three methods fully cover this range. The 125–7500 Hz band also uses 64 mel filters, but frequencies below 125 Hz, which may be useful, are excluded. Additionally, 30 of the 64 mel bands fall outside the presumed useful frequency range. However, because the VGGish network was trained on a YouTube’s video dataset over the 125-7500 Hz frequency range, it is reasonable to investigate whether the VGGish embedding performs better when input mel-spectrogram settings match those used during its training.[...]

From this parametric study about the choice of the initial acoustic space, it seems that no initial acoustic space is significantly better than another. However, as the effect of rain is even more marked on the temporal trajectories using the mel-spectrogram or the 70-2000 Hz VGGish embedding, one of these approaches should be preferred. The similarity between these two initial acoustic spaces means also that 70-2000 Hz VGGish embedding compresses the mel-spectrogram representation without a significant loss of information, from 6400 to 119 features. These two comments oriented our choice to work with the VGGish 70-2000 Hz for the Bora-Bora dataset.”

The rationale and method for changing the analysis time from 0.96s to 1s has been clarified. [L 280-282]

“ For the VGGish embedding, the first 0.96 s of the mel-spectrograms described above (one per second of raw audio, thus the remaining 0.04 s of those spectrograms are not used. See details in S1-Text) are fed into a Convolutional Neural Network (CNN)”

S1-Text [L 58-78]

“In the first step, CoralSoundExplorer segments each raw sound recording into one-second segments (the raw recordings in the Bora-Bora dataset were 60 seconds long). Each one-second sound segment is then projected into an acoustic space. Three different acoustic spaces can be selected: the mel-spectrum, the mel-spectrogram, and the VGGish embedding.[...]

The VGGish acoustic projection takes the first 0.96 seconds of the mel-spectrogram as input to a pre-trained convolutional neural network (CNN): the VGGish network. To ensure consistency across projections and maintain a minimal one-second analysis step, the remaining 0.04 seconds of the mel-spectrogram are excluded.”

The explanation of the frequency band mismatch was addressed in the new part .

241: Mention what VGGish is pre-trained on - a large audio dataset.

We have mentioned it in the new manuscript (L 280-283)

“[..]the mel-spectrograms described above [...] are fed into a Convolutional Neural Network (CNN) pre-trained on a large YouTube’s audio-video dataset named VGGish [73]”

266: Couldn't the reproducibility issue be solved simply by fixing the random seed?

Fixing the random seed for the UMAP process could indeed enable reproducible execution of different sections of *CoralSoundExplorer* because exactly the same UMAPs will be computed and will lead to the same 2/3D UMAP visualization or the same average distances matrix. However, we don’t know *a priori* which random seed will produce the best UMAP representation. In our case, UMAP stochasticity is more a problem of having an averagely good preservation of the data organization's global structure than having reproducible representations.

“The UMAP transformation process is not deterministic and no individual UMAP is a priori better than another. This stochasticity involves differences in point positions between two different UMAPs projections. Metrics calculated directly from these projections or from point distance matrices are also affected by this stochasticity. Thus, to mitigate the random effects of the UMAP algorithm and reduce the uncertainties of the analysis metrics, the UMAP dimension reduction process is repeated.”

275: "UMAP projections": The term "projection" can have a specific mathematical meaning that differs from how UMAP works. UMAP is based on manifold learning, which is slightly different from linear projections like PCA. While "projections" in the context of UMAP is accepted and used, alternative terms like "embeddings" might be more appropriate.

As suggested, UMAP projection(s) occurrences have been replaced by UMAP embedding(s) in the entire new manuscript.

529: "Clusters ... are common at all replicates but are few related to specific daytime hours" is unclear. Is it meant that clusters are weakly/un-related to time of the day?

We clarified the sentence (L598 - 596):

“Fig 15 shows the temporal trajectory of the soundscape at the boat site. During the day, the overall pattern is more difficult to discern than for the other two sites, probably because boat activity is rather chaotic (variation in the number and type of boats, as well as the time of day they sail; Fig 15A). Clusters 1 and 2 (corresponding to day; Fig 15B, 15C, & 15D) are common to all replicates but are unrelated to time of the day. During the late-night hours (from 11 p.m. to approximately 4 a.m.), which correspond to a low boating activity, the temporal trajectories of the 3 replicates all crossed cluster 0. However, the trajectory of replicate 2 deviates from the other two between 10 a.m. and 12 a.m. (Fig 15C), probably highlighting a difference in noise due to marine traffic.”

599: "semi/unsupervised learning methods": What is meant by semi-supervised learning in the proposed method? (See comment about line 618)

We deleted this term in the manuscript. The use of Semi-supervised wasn’t correctly used.

609: I am not familiar with the use of the term "individuals" to refer to data points. Data points, or samples, would sound more natural to me.

As suggested, the word “individuals” has been changed to “data points”

617: Typo: embedding space instead of embedded space

As suggested, we corrected this word in the entire manuscript.

618: "Semi-supervised" typically refers to a form of learning that leverages both labeled and unlabeled data to build better models, especially when labeled data is limited. In this case, however, the term doesn't seem to apply, as there is no learning (i.e., no guided update of model parameters) informed by the labels taking place.

We deleted this term in the manuscript. The use of Semi-supervised wasn’t correctly used.

657: For a given fixed duration, the number of samples per STFT step depends on the sampling rate of the input file. Does the tool resample files to a fixed sampling rate? (See also comment about line 214)

CoralSoundExplorer does not resample audio files. The STFT step is fixed to fs*0.01. Thus the number of STFT steps per second does not depend on the sampling frequency. This has now been made clear in the manuscript (L 275-277):

“The same mel scale as for the mel-spectrum is used but the short time spectrums are computed over 2048 samples with a hop size of 0.01 seconds. Working with a sampling frequency of 44100 Hz leads to a hop size of 441 samples.”

S2-Text [L 68-72]

“the mel-spectrogram was computed over one hundred time steps per one second sound sample, using a 2048-sample STFT Hanning window and a hop size of 0.01s (for a sampling frequency of 44100 Hz this corresponds to a window size of 46.4 ms and a time step of 441 samples). The resulting acoustic space consists of 6400 features (i.e., the number of pixels in the mel-spectrogram).”

750: "'By chance' value" is more commonly referred to as "chance level", without quotes.

We have changed this (now in supplementary section S2-Text) [L 131, 133, 139, Fig S2-Text-14]

752: Typo: Monte Carlo, not Monte Carlos

We have changed this (now in supplementary section S2-Text) [L 133, 135].

796: Sentence ending on line 797 is very unclear. What values could occur?

We have moved the sentence in S2-Text and added additional details [L 150]

“The chance level of the RTA is equal to the number of permutations achieving the correct ranking of the 3 distances (only 1 permutation of the 3 distances is correct) out of the total number of possible permutations of the 3 distances, i.e. 1/P(3,3) = (3!)-1 = 1/6.”

797: "Modality" often means something else. "Class," "label class," or "category" are more common terms for referring to different groups or labels in data classification.

“Category” has been preferred to “modality” in the revised manuscript.

818: "expected" instead of "supposed"

Done (L 130).

Lastly:

Fig 3: There is a mismatch between caption and figure

We have changed the figure 3 [L 228]

While VGGish is an important, general-purpose audio feature extractor, its representation space is optimised for a different set of sounds: VGGish was pre-trained on a large YouTube audio dataset, a data domain different from bioacoustics. In fact, for several biomes and groups of species, BirdNet (<https://doi.org/10.1016/j.ecoinf.2021.101236>) has been shown to be a better embedding space (see <https://ai-2-ase.github.io/papers/35%5cCameraReady.pdf> and <https://doi.org/10.1038/s41598-023-49989-z>). It would be informative for the readers to state the limitations of applying a model trained on YouTube, and what improvements could be expected in the future by the incorporation of a domain-specific large pre-trained embedding model.

As suggested, in the revised manuscript, we have discussed this problem of using a network trained on youtube video (the VGGish training dataset did not contain any coral reef sound and maybe only a few of natural soundscapes recordings) and the consequences on the results given by *CoralSoundExplorer* [L733-739].

“Although different initial acoustic projection spaces produce slightly different results, the overall conclusions regarding distinctions and similarities between recording days, sites, and diurnal/nocturnal periods remain consistent. This result may seem surprising, as VGGish was designed to capture more complex sound structures than raw spectrum and spectrogram. However, the VGGish network was trained on a dataset that likely contained very few, if any, coral reef sounds. Consequently, it may rely primarily on basic spectrogram features to project these soundscape samples..”

We also discussed the opportunities of working with a more domain-specific CNN like BirdNet [L 953-959] and included two of the proposed references.

“A network more dedicated to biological sounds detection in natural recordings, such as birdNet [83] or Perch [84], could provide a more suitable embedding for natural soundscapes study [85], more optimized for animal vocalisations, and thus give more information on the composition of soundscapes.”
